# Cryo-EM structure and resistance landscape of *M. tuberculosis* MmpL3: An emergent therapeutic target

**DOI:** 10.1016/j.str.2021.06.013

**Published:** 2021-10-07

**Authors:** Oliver Adams, Justin C. Deme, Joanne L. Parker, the CRyPTIC Consortium, Philip W. Fowler, Susan M. Lea, Simon Newstead

**Affiliations:** 1 Department of Biochemistry, University of Oxford, Oxford OX1 3QU, UK; 2 The Sir William Dunn School of Pathology, University of Oxford, Oxford OX1 3RE, UK; 3 Central Oxford Structural Molecular Imaging Centre (COSMIC), University of Oxford, Oxford OX1 3RE, UK; 4 Center for Structural Biology, Center for Cancer Research, National Cancer Institute, Frederick, MD 21702-1201, USA; 5 Nuffield Department of Medicine, John Radcliffe Hospital, University of Oxford, Oxford OX3 9DU, UK; 6 National Institute of Health Research (NIHR) Oxford Biomedical Research Centre, John Radcliffe, Oxford OX3 9DU, UK; 7 The Kavli Institute for Nanoscience Discovery, University of Oxford, Oxford OX1 3QU, UK

**Keywords:** tuberculosis, cryo-EM, MmpL3, MmpL, drug resistance, RND transporter, mycolic acid, LMNG, membrane protein, antitubercular

## Abstract

Tuberculosis (TB) is the leading cause of death from a single infectious agent and in 2019 an estimated 10 million people worldwide contracted the disease. Although treatments for TB exist, continual emergence of drug-resistant variants necessitates urgent development of novel antituberculars. An important new target is the lipid transporter MmpL3, which is required for construction of the unique cell envelope that shields *Mycobacterium tuberculosis* (*Mtb*) from the immune system. However, a structural understanding of the mutations in *Mtb* MmpL3 that confer resistance to the many preclinical leads is lacking, hampering efforts to circumvent resistance mechanisms. Here, we present the cryoelectron microscopy structure of *Mtb* MmpL3 and use it to comprehensively analyze the mutational landscape of drug resistance. Our data provide a rational explanation for resistance variants local to the central drug binding site, and also highlight a potential alternative route to resistance operating within the periplasmic domain.

## Highlights

• Cryo-EM structure of *M. tuberculosis* MmpL3 determined at 3.0 Å resolution
• An LMNG molecule within the periplasmic cavity suggests the TMM export pathway
• Comprehensive structural mapping of resistance-conferring MmpL3 variants
• Genome-mined MmpL3 mutations indicate minimal pre-existing resistance

## Graphical abstract

**Figure:**
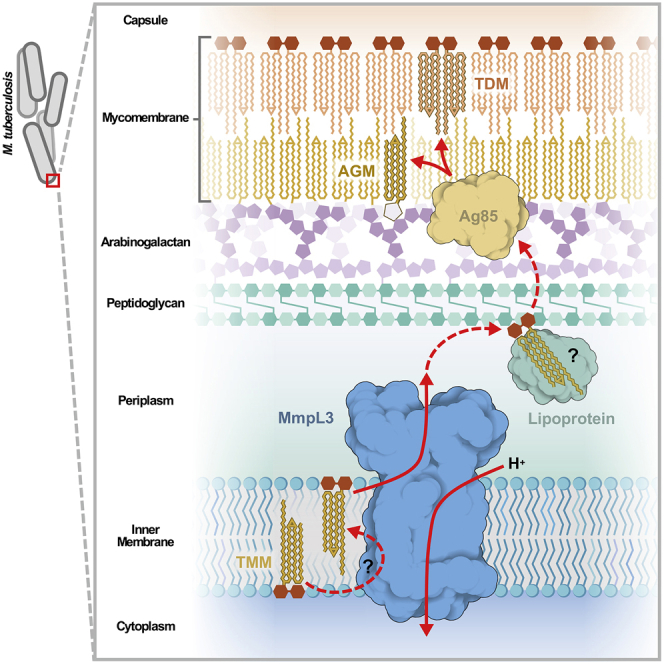

## Teaser

Adams et al. report a structure of the *M. tuberculosis* glycolipid transporter MmpL3, a protein essential to envelope biogenesis and an emergent therapeutic target. The structure reveals the probable lipid export pathway, and mapping of resistance-conferring mutations suggests the existence of multiple routes to preclinical inhibitor evasion.

## Introduction

Tuberculosis (TB) remains the leading cause of death attributable to a single pathogen (World Health Organization, 2020). Compounding this is the emergence and endemic nature of several drug-resistant *Mycobacterium tuberculosis* (*Mtb*) strains (Udwadia et al., 2012; Velayati et al., 2009). Mycobacterial membrane protein large 3 (MmpL3) is part of a vital biosynthetic pathway in *Mycobacterium* species and represents a promising new avenue for novel antitubercular drug development (Rayasam, 2014).

MmpL3 is an essential protein belonging to the resistance-nodulation-division (RND) superfamily of transporters (DeJesus et al., 2017; Domenech et al., 2005). Performing diverse functions throughout all domains of life, the RND superfamily were initially characterized as multi-drug efflux pumps in Gram-negative bacteria (Nikaido, 2018). In Gram-positive mycobacteria the MmpL family predominantly function as endogenous lipid transporters, with many, including MmpL3, involved in the biogenesis of the multi-layered cell envelope (Chalut, 2016; Viljoen et al., 2017). Specifically, MmpL3 exports intracellularly synthesized trehalose monomycolate (TMM) into the periplasm (Grzegorzewicz et al., 2012). TMM transport is essential for the formation of the unique *Mycobacterium* cell envelope that functions as the main physical barrier to drug entry and neutralization by the immune system (Dulberger et al., 2020; Jackson, 2014). The driving force for TMM export is thought to come from the proton-motive force (PMF) established across the inner membrane (Grzegorzewicz et al., 2012; Székely and Cole, 2016), although the precise mechanism by which the PMF drives lipid export remains unclear.

Disruption of envelope mycolation, as induced by small-molecule or genetic inhibition of MmpL3, has repeatedly been shown to be bactericidal (Degiacomi et al., 2017; Li et al., 2016; Shao et al., 2020; Varela et al., 2012). Indeed, the list of preclinical MmpL3 inhibitors is ever-growing, and features compounds with diverse chemical scaffolds (Dupont et al., 2016; De Groote et al., 2018; Grzegorzewicz et al., 2012; Lun et al., 2013; Remuiñán et al., 2013; La Rosa et al., 2012; Stanley et al., 2012; Tahlan et al., 2012). Encouragingly, some have been observed to synergize with existing antituberculars (Li et al., 2017; Nikonenko et al., 2007; Stec et al., 2016) and one, the ethylenediamine SQ109, has completed phase II clinical trials in various countries (Butler and Paterson, 2020; Degiacomi et al., 2020). Significant insights into the interactions between MmpL3 and its inhibitors were recently reported using the *Mycobacterium smegmatis* (*Msmg*) ortholog (Yang et al., 2020; Zhang et al., 2019), which shares 61% sequence identity to *Mtb*. Nevertheless, given the severity of the TB pandemic, the evident susceptibility of MmpL3 to small-molecule inhibitors, and the numerous medicinal chemistry campaigns against it, understanding the structure and biochemical properties of the *Mtb* transporter remains a high priority.

Here, we report the cryoelectron microscopy (cryo-EM) structure of *Mtb* MmpL3 at a resolution of 3.0 Å. We present the most comprehensive structural mapping of MmpL3 variants to date, analyzing over 100 unique resistance-conferring substitutions selected by exposure to preclinical agents. In addition, we examine non-synonymous mutations mined from >45,000 whole-genome sequenced *Mtb* isolates. In conjunction with the recently reported structures of MmpL3 from *Msmg*, our work provides a foundation for structure-based drug design against *Mtb* MmpL3, guided by an enhanced appreciation of the underlying mutational landscape.

## Results and discussion

### Cryo-EM structure of *Mtb* MmpL3

**Figure 1:**
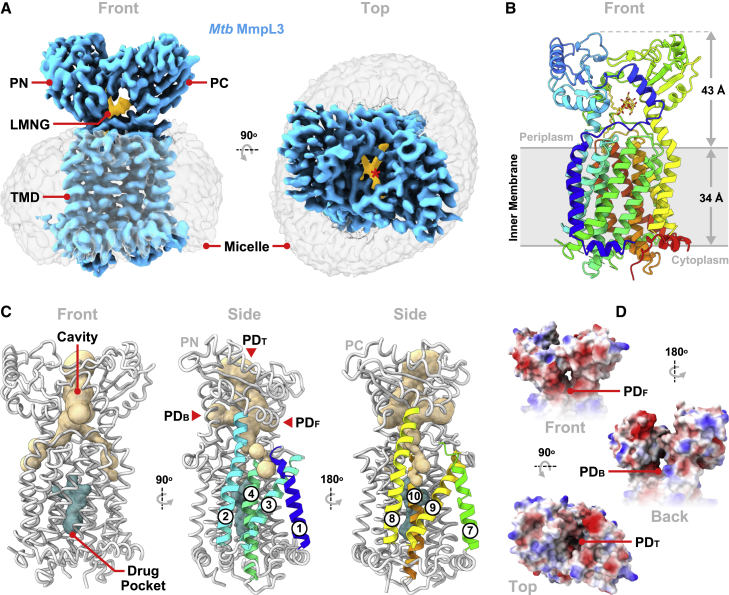
Cryo-EM map and structural analysis of *Mtb* MmpL3 (A) Cryo-EM density of *Mtb* MmpL3_1-753_ as viewed from the membrane plane (front), or perpendicular to it from the periplasm (top). Proteinaceous density is blue (contour level 0.427, sharpened map), the surrounding detergent micelle gray, and a bound LMNG molecule gold (contour level 0.150, unsharpened map). Annotations highlight the transmembrane domain (TMD), and the N-terminal (PN) and C-terminal (PC) lobes of the periplasmic domain (PD). (B) Cartoon representation of the *Mtb* MmpL3_1-753_ atomic model, colored blue (N terminus) to red (C terminus). LMNG is shown as sticks (gold). (C) Licorice representations of *Mtb* MmpL3_1-753_ (gray) accompanied by surface depictions emphasizing its PD cavity network (wheat, calculated using Caver Analyst 2.0 [Jurcik et al., 2018]) and TMD drug binding pocket (teal). The TMs (1–4 and 7–10) lining cavity exits into the outer leaflet are indicated. (D) Electrostatic surface renderings of the MmpL3_1-753_ PD, highlighting the hydrophilic residues gating the labeled PD_F_, PD_B_, and PD_T_ cavity openings. See also Figures S1 and S2, and Table S1.

To obtain the structure of *Mtb* MmpL3 we generated a C-terminally truncated (residues 1–753) construct capable of being overexpressed in *E. coli* (Figure S1A). The intracellular C-terminal domain (CTD) is dispensable for transport, with multiple C-terminal *Mtb* MmpL3 truncations viable *in vivo* (Belardinelli et al., 2016). Despite extensive efforts, MmpL3_1-753_ proved recalcitrant to crystallization. However, cryo-EM imaging of the protein purified in lauryl maltose neopentyl glycol (LMNG) permitted its three-dimensional reconstruction to a resolution of 3.0 Å (Figures S1B–S1E). Our map reveals MmpL3_1-753_ to be monomeric (Figure 1A), consistent with the *Msmg* homolog (Su et al., 2019; Zhang et al., 2019) (Figure S2A). The cryo-EM map was of sufficient quality to confidently model almost the entirety (residues 1–342, 378–752) of the construct (Figures 1B and S1F; Table S1). *Mtb* MmpL3_1-753_ adopts a transmembrane domain (TMD) fold archetypal of RND proteins, consisting of 12 transmembrane helices (TMs) arranged as two sequence-contiguous bundles (TMs 1–6 and 7–12). These are related by a central 2-fold pseudo-symmetry axis running perpendicular to the membrane plane (Figure S2B). The MmpL3_1-753_ TMD is braced against the inner leaflet of the cytoplasmic membrane via three short, lateral, amphipathic α helices, two preceding TMs 1 and 7, with the third leading into the CTD. Characteristic of RND transporters, sizable periplasmic loops connect TMs 1–2 in the N-terminal half of MmpL3_1-753_ (PN, residues 37–166) and TMs 7–8 (PC, residues 415–544) in the C-terminal half of the molecule. Both display an α-β-α-β-α-β topology, with the first α helix of each contributing to the tertiary structure of the adjacent loop. This partial interdigitation of secondary structural elements results in PN and PC clasping one another to give a singular periplasmic domain (PD), capping the TMD and sharing its pseudo-symmetry axis. The N termini of TM2 and TM8 are longer than the other helices and extend into the PD (Figure 1C), which itself protrudes over 40 Å into the periplasmic space.

**Figure 2:**
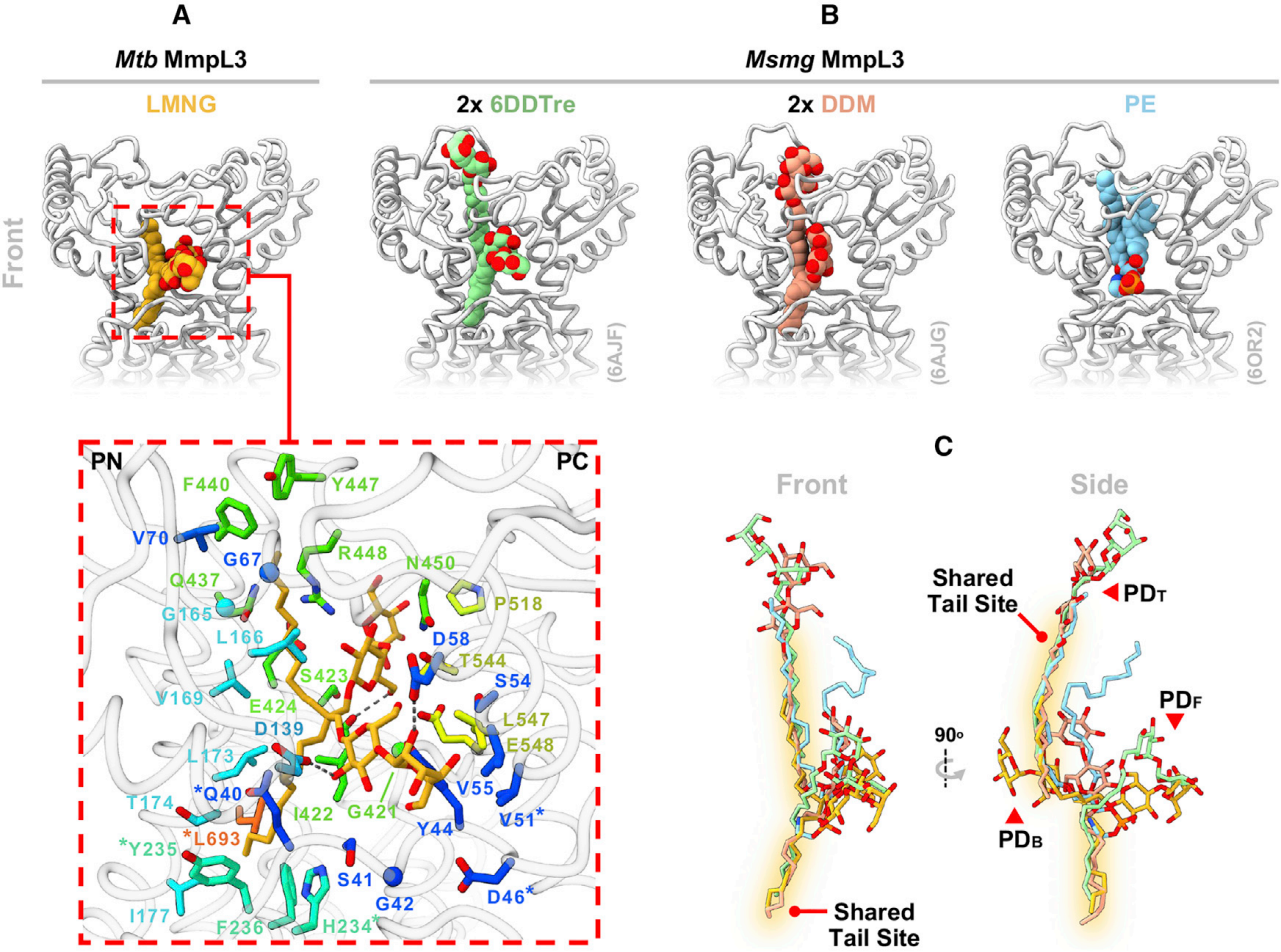
Comparison of MmpL3 periplasmic ligands (A) Close-up of the *Mtb* MmpL3_1-753_ PD LMNG. Residues within 4.5 Å, plus the detergent itself (gold), are shown as sticks; bar glycines shown as balls. Side chains are rainbow colored, as in Figure 1B. Dashed lines denote hydrogen bonds. Asterisks highlight residues conferring inhibitor resistance when mutated. (B) Side-by-side comparison of the 6DDTre (green), DDM (salmon), and PE (blue) molecules co-crystallized within the *Msmg* MmpL3 PD. Ligands are shown as spheres and proteins (gray) in licorice representation. PDB codes are in brackets. (C) Overlay of the *Mtb* and *Msmg* PD ligands, each depicted as sticks and colored as in (A) and (B). Common alkyl chain sites, and the positions of the PD apertures, are annotated.

Similar to structures of the *Msmg* MmpL3 homolog (Su et al., 2019; Zhang et al., 2019), the *Mtb* protein has an extensive cavity enclosed by the PD (Figure 1C). The cavity comprises a central vestibule between the PN and PC lobes, out of which branch two descending channels, each constricted in the vicinity of the PD-TMD “neck.” On opposing faces of the protein these channels exit into the outer leaflet of the inner membrane via grooves bounded by TMs 1–4 or 7–10, respectively. The upper PD chamber likewise possesses three apertures opening into the periplasm from the front (PD_F_), back (PD_B_), and top (PD_T_) of the molecule and each gated by a combination of charged and hydroxyl-containing side chains (Figure 1D). Although the role of this cavity has not yet been fully elucidated, its position within the molecule and relationship to the outer leaflet suggests it forms the conduit through which TMM is extracted into the periplasm, a role supported by site-directed mutagenesis (Belardinelli et al., 2016). Within this cavity a lipid-like species was captured in our structure, most likely the detergent LMNG, which adopts a splayed arrangement bound between the PN and PC subdomains (Figures 1A, 1B, and 2A). LMNG creates a substantial interface, packing against 35 residues (within 4.5 Å) and sitting within the suggested transport path for TMM (Klenotic et al., 2020). The interactions are predominantly contributed by PN, PC, and TM2, but also feature side chains at the N terminus of TM8 and in the loops connecting TMs 3–4 and 11–12 (Figure 2A). Although the LMNG tails point in divergent directions, one halfway down into the channel exiting via TMs 1–4 and the other up toward PD_T_, both are accommodated in pockets partially scaffolded by TM2 aliphatic residues (L166, V169, L173, and I177), and end in aromatic residues (Y235 and F236 or F440 and Y447) provided by nearby secondary structure elements. In contrast, the two LMNG maltose head groups bifurcate to be individually coordinated by residues of the PD_F_ and PD_B_ apertures. In doing so their hydroxyls are hydrogen bonded to side chains of D58 and D139, as well as the backbone carbonyl of I422, leaving the terminal saccharide of each head group to be partly solvent exposed as it emerges through its respective PD opening.

A key question in the mechanism of MmpL3 is how this protein handles the large and complex TMM lipid. The previous *Msmg* MmpL3 structures were co-crystallized with three unique PD occupants (Figure 2B); pairs of the detergents lauryl-6-trehaloside (6DDTre, a structural analog of TMM) and dodecyl maltoside (DDM) (Zhang et al., 2019), as well as a single phosphatidylethanolamine (PE) lipid (Su et al., 2019). Structural comparison with the *Mtb* MmpL3_1-753_-LMNG complex identifies striking similarities in the binding poses of all four ligands (Figure 2C). The pockets embracing the LMNG alkyl chains in our structure are also exploited by 6DDTre and DDM to accommodate their respective hydrophobic tails. PE, in contrast, sits with its shorter heptadecanoate acyl chain in the upper of these two tail sites, leaving its peripheral carbons to extend through PD_T_. The comparison of *Mtb* MmpL3 and *Msmg* MmpL3 suggests a “division of labor” model for PD lipid handling, wherein the central vestibule sequesters the alkyl chains away from the bulk periplasm, potentially into specific pockets (Figure 2C), while the proximate hydrophilic openings (PD_F_, PD_B_, and PD_T_) bind the polar head group, coordinating it through hydrogen bonds and providing it a degree of solvent access. Segmentation of binding would permit accommodation of lipidic cargo in, and subsequent manipulation into, diverse conformations. This flexibility is plausibly essential for translocation of substrates as large as TMM (molecular weight >1.4 kDa, Fujita et al., 2005), and likely underlies the lipid binding promiscuity observed for *Msmg* MmpL3 by native mass spectrometry (Su et al., 2019).

### Mapping MmpL3 mutations

**Figure 3:**
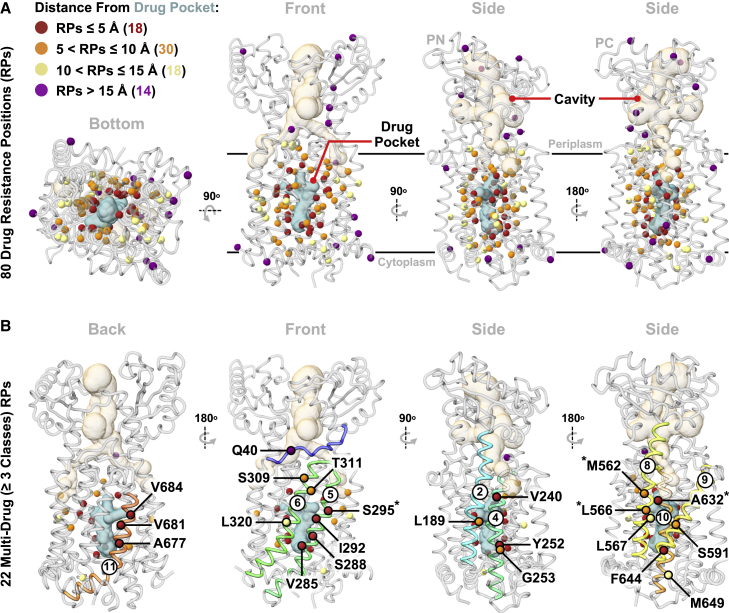
Mapping MmpL3 drug-resistance mutations (A) Eighty resistance positions (RPs) plotted onto *Mtb* MmpL3_1-753_ as balls, color-coded by distance from the universal drug pocket (teal). The PD cavity is also shown (wheat surface). (B) Residues of the 22 RPs causing cross-resistance to a minimum of three classes of MmpL3 inhibitor. Black asterisks denote cross-RPs only observed in *M. abscessus* MmpL3. See also Figures S3 and S4, and Table S2.

MmpL3 is the target of many preclinical antituberculars (Degiacomi et al., 2020). These compounds target a conserved central pocket within the TMD that sits at the interface between TMs 4 and 5 and 10 and 11 (Figures 1C and S4B) and incorporates the Asp-Tyr interaction network (Figure S2C) proposed to function in coupling TMM transport to the PMF (Bernut et al., 2016; Yang et al., 2020; Zhang et al., 2019). Antitubercular agents have been predominantly discovered by high-throughput whole-cell phenotypic screening against sizable chemical libraries; with targets for active compounds inferred by their mutation in whole-genome-sequenced resistant isolates (Goldman, 2013). Consequently, there exists a substantial repository of drug-resistant MmpL3 variants in the literature, which we collated and mapped onto our MmpL3_1-753_ model. We aggregated 112 unique resistance-conferring mutations occurring at 83 different positions in MmpL3, 21 of which differ between the *Msmg* and *Mtb* homologs (Table S2). Of the 112 mutations, 109 variants at 80 positions were mappable onto our *Mtb* MmpL3_1-753_ structure (Figure 3A). The remaining positions populated the unresolved inter-repeat linker. Most mutations were described in *Mtb* (57 positions), but to provide a more complete picture of the resistance landscape we also back-mapped variants originally identified in *Msmg* (11 positions)*, M. bovis* (13 positions), and *M. abscessus* (19 positions) by sequence alignment (Figure S4A).

Of the 83 resistance positions (RPs) identified, ~90% reside in the TMD and 60% are within 10 Å of the drug pocket (Figure 3A). Such clustering suggests that the dominant route to resistance is through mutation of residues defining the 2-fold pseudo-symmetry axis relating the two six-helix bundles of the TMD. These mutations likely weaken the affinity of antitubercular drugs targeting the transmembrane site, as has been demonstrated for a handful of *Msmg* MmpL3 variants by MST (Zhang et al., 2019). This conclusion is also consistent with proposals that the magnitude and spectrum of the resistance phenotype given by an MmpL3 mutant correlates with its proximity to this pocket (Williams et al., 2019). However, our analysis reveals that a significant proportion of RPs are located >10 Å away from the drug pocket, including some within the LMNG binding site (asterisks in Figure 2A), suggesting that there is at least one additional, and as yet uncharacterized, MmpL3 resistance mechanism; likely involving the PD and its cavity. Future studies exploring the role of the PD within the mechanism of MmpL3 will be required to uncover how these mutations circumvent the action of drugs targeting the TMD.

To further dissect the resistance landscape, we categorized the RPs by the nine established MmpL3 inhibitor classes, as well as a tenth miscellaneous grouping of orphan scaffolds (Figure S3). All the scaffolds retained RP clustering around the common drug binding pocket, corroborating its use by classes yet to be structurally characterized in complex with MmpL3. Armed with our RP mapping data we undertook a finer analysis with respect to the drug binding poses resolved in the *Msmg* structures. This highlighted that class-specific differences in RP distribution can be attributed to subtleties in the interactions each makes with MmpL3. The most obvious example are the pyrrole/pyrazole class inhibitors, where RPs are enriched around a subsite (residues L243, V681, V684, A685, and L703) unused in the binding of other structurally characterized scaffolds (Figure S3A) (Zhang et al., 2019). Equally, the differences apparent between indolecarboxamides (Figure S3C) and spirocycles (Figure S3G) agrees with observations that SPIRO, an example of the latter, makes fewer interactions at the top of the shared inhibitor pocket, but more at the bottom, relative to NITD-349, a member of the former family (Yang et al., 2020). Thus, despite significant overlap, there is clear differentiation in the pattern of RPs selected by each drug class.

A greater understanding of the MmpL3 mutations capable of conferring resistance to multiple scaffolds is critical to design of agents less easily inducing resistance. Such knowledge, for example, could direct medicinal chemistry efforts toward minimizing lead interactions with the offending residues. We therefore filtered for RPs where variants have been associated with resistance to at least three MmpL3 inhibitor classes (Figure 3B). Twenty-two RPs were identified, all except Q40 fell within the TMD and 50% were within 5 Å of the common binding pocket. Mutations of Q40, which resides in the linker joining TM1 to the first PN helix and is >15 Å from the drug binding site, almost certainly act by the aforementioned “alternative” resistance mechanism. Consistently, the Q40R variant failed to alter *Msmg* MmpL3 affinity for inhibitors as measured by MST (Zhang et al., 2019). Q40 lines the MmpL3_1-753_ channel exiting via TMs 1–4, within the LMNG binding site, and has thus far only been reported to confer resistance phenotypes on mutation to cationic amino acids (R or H, Table S2). It represents an important site for future biochemical studies.

Structural interpretation of the physiochemical changes introduced by variants at the other 21 cross-RPs identifies a number of plausible explanations for their phenotypes. A facile route to resistance, with minimal fold disruption, would be conservative binding site mutations that alter side chain size, thereby impairing steric complementarity. This mechanism is apparent for three *Mtb* drug-interacting valines (V240, V285, and V684) that mutate to smaller residues (A or G, Table S2) to achieve multi-class resistance. The reverse mechanism, enlarging side chains to promote steric clashes with complexed inhibitors, is employed at two drug-interacting sites, where alanine side chains (A632 and A677) are substituted for bulkier aliphatics or aromatics (Table S2). Modulation of residue hydrogen bond capacity is another route to resistance, either through loss (T311I, S591I), gain (I292S/T, L566S), or exchange (S288T) of hydroxyl-containing side chains (Table S2). Further away from the shared drug pocket many of the substitutions at cross-RPs result in dramatic chemical perturbations, in line with both their presumed action at a distance, and a diminished capacity to collaterally incapacitate the proton-relay system. These less-conservative variants either introduce additional charge into the TMD (L189R and G253E) or supply intra-TM prolines (S295P, L320P, and L567P, Table S2), which is conformationally disruptive. Interestingly, four of the 22 cross-RPs have so far only been observed in *M. abscessus* (asterisks in Figure 3B). This suggests that MmpL3 orthologs may preferentially mutate at different residues to facilitate drug escape, an important caveat if future antitubercular MmpL3 inhibitors are to be repurposed for treatment of non-tuberculous mycobacterial infections (Li et al., 2018).

A large number of *Mtb* genomes (45,878), mostly originating from clinical samples, are available from the European Nucleotide Archive (ENA). By analyzing these with respect to our resistance catalog (Table S2) one can estimate how much environmental resistance any new antibiotics targeting MmpL3 are likely to encounter. Consistent with its essential nature, no MmpL3 mutations were detected in the majority of genomes; 3,890 (8.5%) out of the 45,878 samples harbored 7,475 non-synonymous mutations (Table S3). In total, 597 distinct amino acid substitutions were identified; however, the majority (422, 71%) were seen three or fewer times. Only 25 mutations were detected in 30 or more samples and accounted for 5,374 (72%) of the non-synonymous changes.

**Figure 4:**
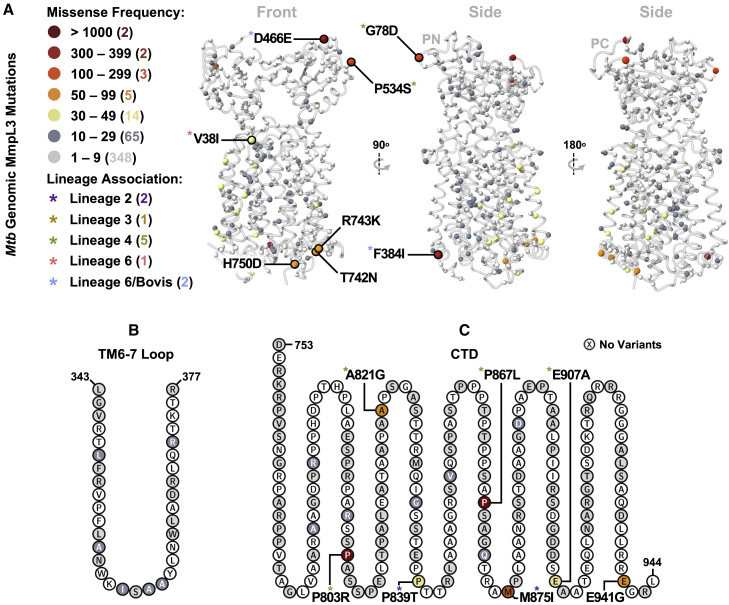
Wider non-synonymous landscape of MmpL3 (A) Structural mapping of MmpL3 variants mined from over 45,000 *Mtb* genomes, represented by balls and color-coded by mutational frequency. The most abundant substitutions, and/or those with apparent *Mtb* lineage association (colored asterisks) are labeled. (B and C) (B) Incidence of non-synonymous mutations in the TM6-7 loop, and (C) C-terminal domain. See also Figure S4 and Table S3.

These 25 mutations are found throughout the protein (Figure 4) and none were identified by the *in vitro* resistance studies (Table S2). While we cannot conclude that they do not confer resistance, one can infer that it is unlikely. As may be expected, the chemical perturbation introduced by these variants tends to be smaller in the TMD than in the CTD, mirroring domain conservation (Figures S4B–S4D). A subset also appear specific to particular *Mtb* lineages (asterisks in Figure 4), suggesting that they are ancestral (Comas et al., 2013). Conversely, the lack of abundant non-lineage defining mutations (Table S3) implies there is currently little evolutionary pressure on the *mmpL3* gene and/or non-synonymous mutations tend to be deleterious.

The most prevalent resistance-conferring mutation in the genome-mined dataset, V210A, occurs in just 21 samples. Although 16 of these are found in but three studies (Colangeli et al., 2018; Guerra-Assuncąõ et al., 2015; Pankhurst et al., 2016), all three studies sequence clinical samples that can reasonably be expected not to have been exposed to any of the novel MmpL3 inhibitors under development. A further 12 mutations identified by experiment as conferring resistance are present in an additional 32 samples in the ENA (Table S3), but only three (G628A, Y252C, and I585V) are found in more than five isolates. Simply counting these samples, and bearing in mind that the *Mtb* genomes deposited in the ENA are likely to be enriched for resistance, we arrive at 0.1% as an upper limit for the prevalence of pre-existing resistance mutations in MmpL3. This low percentage provides evidence that MmpL3 inhibitors, once licensed, are unlikely to encounter resistant variants in the environment.

In summary, our results provide a structural model of *Mtb* MmpL3, which, combined with our genetic analyses, provides a robust framework for antitubercular development against this essential protein.

## Members of the CRyPTIC Consortium

Derrick W. Crook, Timothy E.A. Peto, A. Sarah Walker, Sarah J. Hoosdally, Ana L. Gibertoni Cruz, Joshua Carter, Alice Brankin, Sarah Earle, Samaneh Kouchaki, Alexander S. Lachapelle, Yang Yang, Timothy M. Walker, Philip W. Fowler, Daniel Wilson, and David A. Clifton, University of Oxford; Zamin Iqbal, Martin Hunt, Kerri M. Malone, Penelope Wintringer, Brice Letcher, and Jeff Knaggs, European Bioinformatics Institute; Daniela M. Cirillo, Emanuele Borroni, Simone Battaglia, Arash Ghodousi, Andrea Spitaleri, and Andrea Cabibbe, Emerging Bacterial Pathogens Unit, IRCCS San Raffaele Scientific Institute, Milan; Sabira Tahseen, National Tuberculosis Control Program Pakistan, Islamabad; Kayzad Nilgiriwala and Sanchi Shah, The Foundation for Medical Research, Mumbai; Camilla Rodrigues, Priti Kambli, Utkarsha Surve, and Rukhsar Khot, P.D. Hinduja National Hospital and Medical Research Center, Mumbai; Stefan Niemann, Thomas Kohl, and Matthias Merker, Research Center Borstel; Harald Hoffmann, Katharina Todt, and Sara Plesnik, Institute of Microbiology and Laboratory Medicine, IML red, Gauting; Nazir Ismail, Shaheed Vally Omar, Lavania Joseph Dumisani Ngcamu, Nana Okozi, and Shen Yuan Yao, National Institute for Communicable Diseases, Johannesburg; Guy Thwaites, Thuong Nguyen Thuy Thuong, Nhung Hoang Ngoc, and Vijay Srinivasan, Oxford University Clinical Research Unit, Ho Chi Minh City; David Moore, Jorge Coronel, and Walter Solano, London School of Hygiene and Tropical Medicine and Universidad Peruana Cayetano Heredia, Lima; George F. Gao, Guangxue He, Yanlin Zhao, Aijing Ma, and Chunfa Liu, China CDC, Beijing; Baoli Zhu, Institute of Microbiology, CAS, Beijing; Ian Laurenson and Pauline Claxton, Scottish Mycobacteria Reference Laboratory, Edinburgh; Robert J. Wilkinson, University of Cape Town, Imperial College London, and Francis Crick Institute; Anastasia Koch, University of Cape Town; Ajit Lalvani, Imperial College London; James Posey, CDC Atlanta; Jennifer Gardy, University of British Columbia; Jim Werngren, Public Health Agency of Sweden; Nicholas Paton, National University of Singapore; Ruwen Jou, Mei-Hua Wu, and Yu-Xin Xiao, CDC Taiwan; Lucilaine Ferrazoli, Rosangela Siqueira de Oliveira, and Juliana Maira Watanabe Pinhata, Institute Adolfo Lutz, São Paulo; James Millard, Africa Health Research Institute, Durban; Rob Warren, University of Stellenbosch, Cape Town; Annelies Van Rie, University of Antwerp; Simon Grandjean Lapierre, Marie-Sylvianne Rabodoarivelo, and Niaina Rakotosamimanana, Institut Pasteur de Madagascar; Camus Nimmo, University College London; Kimberlee Musser and Vincent Escuyer, Wadsworth Center, New York; Ted Cohen, Yale University; E. Grace Smith, Priti Rathod, Lisa Jarrett, and Daniela Matias, Public Health England, Birmingham.

## **Supporting citations**

The following references appear in the supplemental information: Dupont et al., 2019; Foss et al., 2016; Grover et al., 2021; Ioerger et al., 2013; Korycka-Machała et al., 2019; Kozikowski et al., 2017; Li et al., 2019; Li et al., 2020; McNeil et al., 2017; McNeil et al., 2020; Molin et al., 2019; Pandya et al., 2019; Poce et al., 2013; Poce et al., 2019; Rao et al., 2013; Raynaud et al., 2020; Shetty et al., 2018; Tantry et al., 2015; Trofimov et al., 2018; Zheng et al., 2018.

## STAR★Methods

### Key resources table

**Table:** 

REAGENT or RESOURCE	SOURCE	IDENTIFIER
**Bacterial and virus strains**		

*E. coli* OmniMAX	Invitrogen	Cat# C854003
*E. coli* C43(DE3)	Lucigen	Cat# 60446-1

**Chemicals, peptides, and recombinant proteins**		

Lauryl Maltose Neopentyl Glycol (LMNG)	Anatrace	Cat# NG310
TEV Protease	Produced In-house	N/A

**Critical commercial assays**		

HisPur-Ni-NTA Resin	Thermo Fisher Scientific	Cat# 88221
HisTrap Column	Cytiva	Cat# 17-5255-01
Superdex 200 10/300 Increase GL Column	Cytiva	Cat# 28-9909-44

**Deposited data**		

*Mtb* MmpL3_1-753_ Cryo-EM Volume	This Paper	EMDB code: EMD-12604
*Mtb* MmpL3_1-753_ Atomic Coordinates	This Paper	PDB code: 7NVH
*Msmg* MmpL3_1-773_ Atomic Coordinates	Su et al., 2019	PDB code: 6OR2

**Oligonucleotides**		

Primer: NdeI MmpL3_1-753_ Forward GGAATTCCATATGTTTGCATGGT GGGGTCG	This Paper	N/A
Primer: BamHI MmpL3_1-753_ Reverse ACTGGGATCCATCAGGCAGATGA ATTTCACCCAGGCC	This Paper	N/A

**Recombinant DNA**		

Plasmid: pMA-T-MmpL3_1-944_	Invitrogen GeneArt	N/A
Plasmid: pWaldo-GFPd	Drew et al., 2001	N/A
Plasmid: pWaldo-GFPd-MmpL3_1-753_	This Paper	N/A

**Software and algorithms**		

SIMPLE 3.0	Caesar et al., 2020	https://simplecryoem.com/
cryoSPARC (v.2.15.0)	Punjani et al., 2017	https://cryosparc.com/
UCSF pyem (v.0.5)	Asarnow et al., 2019	https://zenodo.org/record/3576630
RELION 3.1.0	Zivanov et al., 2020	https://www3.mrc-lmb.cam.ac.uk/relion/index.php/Main_Page
Phyre2 Server	Kelley et al., 2015	http://www.sbg.bio.ic.ac.uk/phyre2/html/page.cgi?id=index
Coot (v.0.9.2-pre)	Emsley et al., 2010	https://www2.mrc-lmb.cam.ac.uk/personal/pemsley/coot/
PHENIX (v.1.18.2)	Liebschner et al., 2019	http://www.phenix-online.org/
UCSF Chimera (v.1.13.1)	Pettersen et al., 2004	https://www.cgl.ucsf.edu/chimera/
UCSF ChimeraX (v.1.1)	Pettersen et al., 2021	https://www.rbvi.ucsf.edu/chimerax/
CAVER Analyst 2.0	Jurcik et al., 2018	http://www.caver.cz/
ConSurf Server	Ashkenazy et al., 2016	https://consurf.tau.ac.il/
T-Coffee	Madeira et al., 2019	https://www.ebi.ac.uk/Tools/msa/tcoffee/
ChemDraw Professional (v.17.0.0.206)	PerkinElmer Informatics	https://perkinelmerinformatics.com/products/research/chemdraw/
Illustrate	Goodsell et al., 2019	https://ccsb.scripps.edu/illustrate/
T_E_Xshade	Beitz, 2000a	https://ctan.org/pkg/texshade
T_E_Xtopo	Beitz, 2000b	https://ctan.org/pkg/textopo
Mykrobe	Hunt et al., 2019	https://www.mykrobe.com/

**Other**		

Vivaspin-20 (50 kDa MWCO) Concentrator	Sartorius	Cat# VS2032
Vivaspin-500 (50 kDa MWCO) Concentrator	Sartorius	Cat# VS0132
Quantifoil 300 Mesh Au R1.2/1.3 Grids	Agar Scientific	Cat# AGS143-8

### Resource availability

#### Lead contact

Further information and requests for resources and reagents should be directed to and will be fulfilled by the lead contact, Simon Newstead (simon.newstead@bioch.ox.ac.uk).

#### Materials availability

All unique reagents generated in this study are available from the lead contact upon reasonable request.

#### Data and code availability

The cryo-EM volume has been deposited in the Electron Microscopy DataBank (EMDB) with accession code EMDB-12604, and the atomic coordinates have been deposited in the Protein DataBank (PDB) with accession code 7NVH. The mutational data underlying Figures 3 and 4 are provided in Tables S2 and S3 respectively. This paper does not report original code. Any additional information required to reanalyze the data reported is available from the lead contact upon request.

### Experimental model and subject details

Plasmid propagation and clone recovery were performed using OmniMAX *E. coli* cells (Invitrogen) plated on Luria Broth (LB) agar (37°C) or inoculated in LB liquid media (37°C, 180 RPM); both grown overnight in the presence of a selective antibiotic. Recombinant *Mtb* MmpL3_1-753_ was produced in *E. coli* C43(DE3) cells (Lucigen), cultured in Terrific Broth supplemented with kanamycin (50 μg mL^−1^) for ~18 hr (25°C, 215 RPM) following IPTG induction (final concentration 0.4 mM) at an OD_600_ ~0.6–0.8.

### Method details

#### *Mtb* MmpL3_1-753_ molecular cloning & purification

An *E. coli* codon-optimised version of the gene (*Rv0206c*) encoding full-length (residues 1–944) *Mtb* MmpL3 (Uniprot ID: P9WJV5) was synthesised by Invitrogen GeneArt services, provided in the vector pMA-T. Using the primers detailed in the Key Resources Table, a truncated open reading frame encoding only residues 1–753 of *Mtb* MmpL3 was amplified and then restriction sub-cloned into IPTG-inducible vector pWaldo-GFPd (Drew et al., 2001; Waldo et al., 1999); placing it in-frame with a C-terminal TEV-GFP-His_8_ affinity-tag. MmpL3_1-753_ expression in *E. coli* and purification to homogeneity, by Ni^2+^-affinity chromatography followed by SEC, was achieved using standard protocols (Drew et al., 2005). All purification steps were undertaken at 4°C. Briefly, thawed membranes were first resuspended in 1X PBS supplemented with 150 mM NaCl, prior to solubilization in 1% (w/v) LMNG (Anatrace) under gentle agitation for 1.5 hr. Non-solubilized material was removed by ultracentrifugation (>200,000 g, 1 hr), and then imidazole to a final concentration of 15 mM was added to the recovered supernatant. Protein in this was bound to HisPur-Ni-NTA resin (Thermo Fisher Scientific) in batch, by incubation for ~2 hr 45 min with stirring. The resin, packed into a glass econo-column (BioRad), was next sequentially washed with 10 column volumes (CVs) of purification buffer (1x PBS, 150 mM NaCl, 0.1% (w/v) LMNG) containing 15 mM imidazole, and 15 CVs supplemented with 30 mM imidazole. MmpL3_1-753_ was finally eluted in 5 CVs of purification buffer featuring 250 mM imidazole. After TEV protease addition, the protein was dialyzed overnight in gel filtration buffer (20 mM Tris pH 7.5, 150 mM NaCl, 0.003% (w/v) LMNG). The next day, dialysate was passed through a 5 mL HisTrap column (Cytiva) to remove both the TEV protease and liberated GFP-His_8_ affinity-tag. The resulting pure MmpL_1-753_ was spin concentrated using a Vivaspin-20 (50 kDa MWCO, Sartorius), prior to SEC polishing on a Superdex 200 10/300 Increase GL column (Cytiva) pre-equilibrated in gel filtration buffer. For cryo-EM, SEC fractions of interest were immediately pooled, spin concentrated to 2 mg mL^−1^ in a Vivaspin-500 (50 kDa MWCO, Sartorius), and the protein then kept on ice until grid preparation the same day. MmpL3_1-753_ purity was assessed throughout purification by analysis of samples loaded on 10% SDS-PAGE gels stained with Coomassie blue (InstantBlue, Expedeon).

#### Cryo-EM sample preparation & imaging

A 4 μL aliquot of pure, LMNG-solubilized, MmpL3_1-753_ (2 mg mL^−1^) was dispensed onto the surface of a freshly glow-discharged holey carbon-coated grid (Quantifoil 300 mesh, Au R1.2/1.3, Agar Scientific). After allowing 10 s for adsorption, the grid was blotted for 3 s (blot force −5, 100% humidity, 8°C) and then flash-frozen by plunging into liquid ethane using a Vitrobot Mark IV (Thermo Fisher Scientific). Data were gathered in counting super-resolution mode on a 300 kV Titan Krios G3 cryo-TEM (Thermo Fisher Scientific) equipped with a BioQuantum Imaging Filter (Gatan) and K3 Direct Electron Detector (Gatan). Using a pixel size 0.832 Å, 15,020 movies spanning a defocus range of −3.0 to −0.5 μm were collected; each with a total dose of 58.2 e−/Å^2^, spread across 40 fractions, over an exposure time of 2.80 s. A representative micrograph is shown in Figure S1B.

#### Cryo-EM data processing

A schematic summarising the MmpL3_1-753_ cryo-EM data processing workflow is provided in Figure S1C. Patched (15 x 10) motion correction, dose weighting, contrast transfer function (CTF) estimation, and particle autopicking were all implemented on-the-fly using SIMPLE 3.0 (Caesar et al., 2020). This yielded 7,764,838 particles, extracted in 256 × 256-pixel boxes, which were then subject to two rounds of reference-free 2D classification; the first on-the-fly within SIMPLE 3.0, using cluster2D-stream, and the second in cryoSPARC (v.2.15.0, Punjani et al., 2017). Discarding poor-quality classes after each round left 1,564,980 particles for downstream processing, which was performed predominantly in cryoSPARC. The particle subset was first subject to multi-class (k = 4) *ab initio* model generation, without symmetry imposition (i.e. in C1). This generated a single featureful volume, displaying the expected MmpL3_1-753_ domain organization, composed of 758,680 particles. Subsequently passing these through two rounds of non-uniform refinement (Punjani et al., 2020), interspersed by Bayesian particle polishing (Zivanov et al., 2019) undertaken in RELION 3.1.0 (Zivanov et al., 2020) within 320 × 320-pixel boxes, gave a 3.4 Å map. The requisite STAR file for this polishing was generated using the csparc2star.py script from UCSF pyem (Asarnow et al., 2019). Further heterogeneous refinement of the volume against the previous four *ab initio* classes recovered 562,667 particles, whose non-uniform refinement returned a 3.3 Å reconstruction. A final round of reference-free 2D classification was employed to remove any remaining suboptimal particles, resulting in a stack retaining the best 414,082. Successive non-uniform, beamtilt, and local refinement of these yielded the 3.0 Å map of MmpL3_1-753_ deposited in the EMDB under accession code EMD-12604. Throughout processing all refinement reference maps were lowpass filtered to 8 Å. Gold-standard Fourier shell correlations (FSC) using the 0.143 criterion, as well as local resolution estimations, were calculated in RELION 3.1.0 (Figures S1D and S1E).

#### Model building & refinement

Atomic model building began by threading the *Mtb* MmpL3_1-753_ sequence onto a previously reported crystal structure of the *Msmg* orthologue (PDB code: 6OR2, Su et al., 2019), using the Phyre2 web server (Kelley et al., 2015). The threaded structure was then docked into the 3.0 Å cryo-EM volume in UCSF Chimera (v.1.13.1, Pettersen et al., 2004), prior to further rigid body fitting in Coot (v.0.9.2-pre, Emsley et al., 2010). Within the latter program the model underwent manual real-space refinement (RSR), regions lacking unambiguous density were removed (residues 343–377, 753), and an LMNG molecule was built into the splayed species occupying the PD chamber. To generate the final model, detailed in Table S1 and deposited in the PDB under accession code 7NVH, the MmpL3_1-753_-detergent complex underwent further rounds of RSR in PHENIX (v.1.18.2, Liebschner et al., 2019) with secondary structure, rotamer, ligand, and Ramachandran restraints applied, alongside additional minor adjustments in Coot. The globally sharpened map, output from cryoSPARC local refinement, was used for all rounds of RSR in both Coot and PHENIX. Model validity was assessed by MolProbity (Williams et al., 2018) executed within the latter suite. The density fit for modeled TM side chains and the LMNG ligand are shown in Figure S1F.

#### Structural analysis & data Visualisation

The MmpL3_1-753_ cavity was calculated with Caver Analyst 2.0 (Jurcik et al., 2018) using default parameters. Conservation analysis was performed on the ConSurf server (Ashkenazy et al., 2016). The MmpL3 ortholog sequence alignment was generated by T-Coffee (Madeira et al., 2019), and typeset with T_E_Xshade (Beitz, 2000a). All structural figures were composed in UCSF Chimera (v.1.13.1, Pettersen et al., 2004) or UCSF ChimeraX (v.1.1, Pettersen et al., 2021). Primary sequence representations of the structurally unresolved regions of *Mtb* MmpL3 were made with T_E_Xtopo (Beitz, 2000b). Antitubercular skeletal formulas were prepared in ChemDraw Professional (v.17.0.0.206), and the protein elements of the graphical abstract were produced using Illustrate (Goodsell et al., 2019).

#### *Mtb* MmpL3 mutation collation

Variants of *Mtb* MmpL3 observed to confer resistance to one or more preclinical agents were extracted from 29 publications. These were then manually curated to give Table S2, which includes references to all the original studies. Mutations identified in mycobacterial species other than *Mtb* were back-mapped by means of sequence alignments between the relevant MmpL3 orthologs. The broader mutational landscape of *Mtb* MmpL3 was sampled by retrieving all *Mtb* genomes deposited in the ENA at the time of study, together with those reported in Walker et al. (2015) and The CRyPTIC Consortium and the 100,000 Genomes Project, 2018. Totalling 45,878, the majority of these genomes underwent *Mtb* lineage classification using Mykrobe (Hunt et al., 2019) and the short reads were mapped onto version 3 of the H37Rv *Mtb* reference genome (NC_000962.3) and variants called using Clockwork v.0.8.3 as described elsewhere (The CRyPTIC Consortium, 2021). The CRyPTIC data warehouse was then interrogated for all non-synonymous substitutions in the MmpL3 open reading frame. The resultant 7,475 mutations, and any lineage associations of the most abundant, are compiled in Table S3.

### Quantification and statistical analysis

Cryo-EM reconstruction and model building of *Mtb* MmpL3_1-753_ was performed as described in the relevant ‘Method details’ sections, employing the software packages SIMPLE 3.0, cryoSPARC (v.2.15.0), RELION 3.1.0, Coot (v.0.9.2-pre), and PHENIX (v.1.18.2) as noted in the Key Resources Table. Final model statistics are given in Table S1.

## Supplemental information

Document S1. Figures S1–S4 and Table S1

Table S2. Curated MmpL3 drug-resistance mutations, related to Figures 3 and S3 Table collating resistance-conferring MmpL3 mutations extracted from the literature, representing a total of 112 unique single amino acid substitutions at 83 residues. The preclinical agents, and associated inhibitor classes, affected by each variant are listed, along with the mycobacterial species the mutation was identified in, and a reference to the source study. Variants pooled from species other than *Mtb* are back-mapped, with the original mutation also listed. In only a single case did back-mapping produce a synonymous mutation (see L291).

Table S3. Genome-mined non-synonymous *Mtb* MmpL3 substitutions, related to Figure 4 Table of 7,475 non-synonymous mutations detected in the *Mtb* MmpL3 open reading frame, mined from 45,878 *Mtb* genomes. These are distributed over 597 unique substitutions at 439 different residues. The cumulative mutational frequency per position, as well as any variants with an *Mtb* lineage association as assigned by Mykrobe (Hunt et al., 2019), are also provided.

Document S2. Article plus supplemental information
