# Supplementary material for: Cryo-EM structure and resistance landscape of M. tuberculosis MmpL3: An emergent therapeutic target
Source: Structure. 2021 Oct 7;29(10):1182–1191.e4. doi: 10.1016/j.str.2021.06.013 (PMC8752444; doi:10.1016/j.str.2021.06.013)
Supplement: Document S1. Figures S1–S4 and Table S1 [file mmc1.pdf]

Structure, Volume *29*

Supplemental Information

Cryo-EM structure and resistance landscape of *M. tuberculosis* MmpL3: An emergent therapeutic target

Oliver Adams, Justin C. Deme, Joanne L. Parker, the CRyPTIC Consortium, Philip W. Fowler, Susan M. Lea, and Simon Newstead

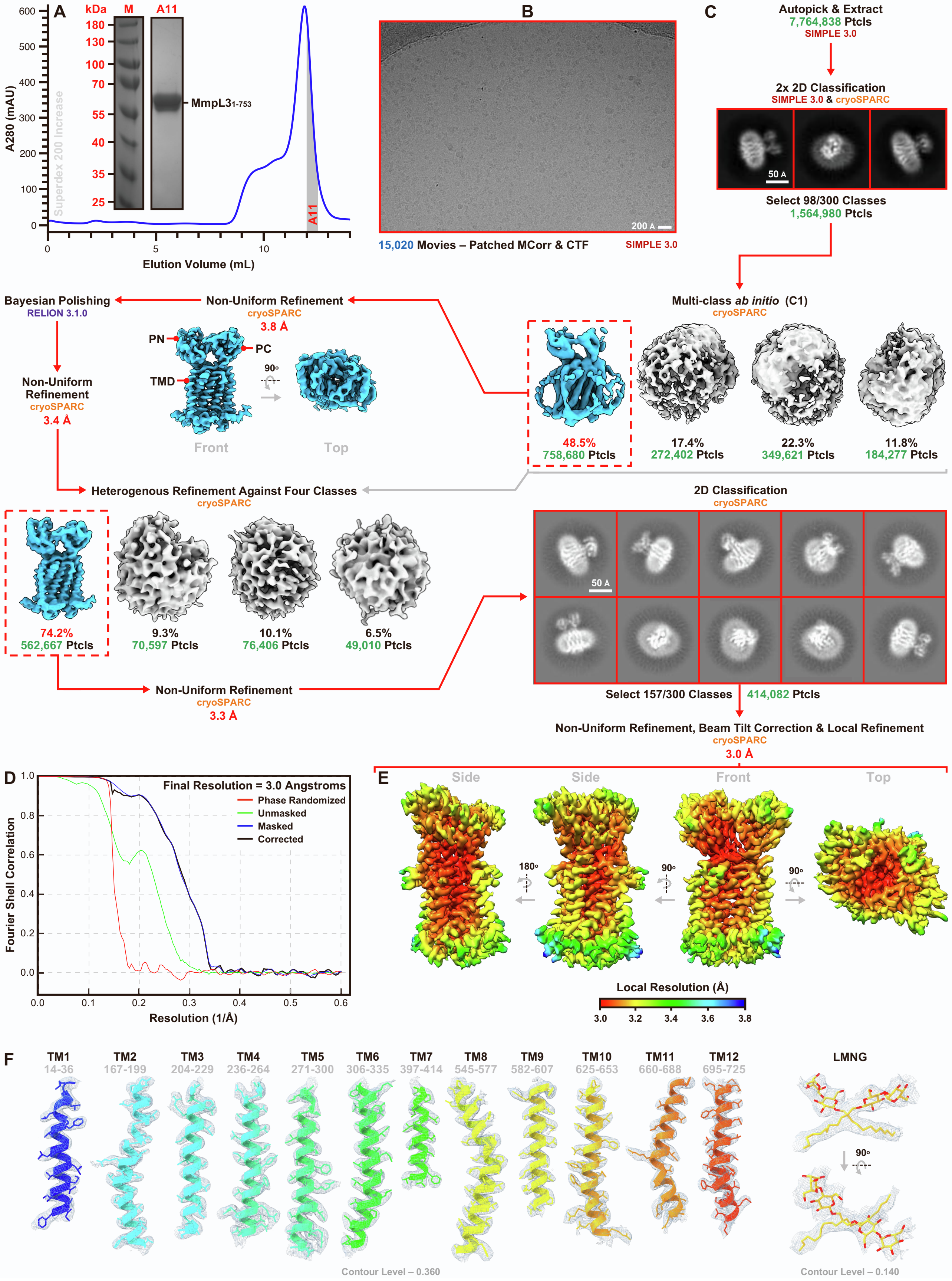

Figure S1. *Mtb* MmpL3$_{1\text{-}753}$ **Purification, Cryo-EM Workflow, Map Resolution and Model Density Fit, Related to Figure 1 and Table S1**

(A) Superdex 200 10/300 Increase GL profile recovered on size-exclusion chromatography (SEC) polishing of LMNG purified *Mtb* $MmpL3_{1-753}$ for cryo-EM studies. The grey bar denotes the SEC fraction ("A11") concentrated for imaging. The same material is analyzed on the inset Coomassie-stained SDS-PAGE gel lane, which confirms purification to homogeneity. Absorption at 280 nm (A280) is in milli-absorbance units (mAU). **(B)** Representative micrograph of the *Mtb* $MmpL3_{1-753}$ sample (2 mg mL^{-1}) collected for cryo-EM structure determination. Scale bar is 200 Å. **(C)** *Mtb* $MmpL3_{1-753}$ cryo-EM data processing workflow. The software package(s) employed at each stage are indicated. Scale bars are 50 Å. **(D)** Fourier shell correlation (FSC) plot for the final *Mtb* $MmpL3_{1-753}$ reconstruction. The stated global resolution is reported at the gold-standard 0.143 cut-off. **(E)** Local resolution estimates for the sharpened *Mtb* $MmpL3_{1-753}$ map, viewed from four directions. Micellar density is hidden. **(F)** Density fit for the 12 modelled *Mtb* $MmpL3_{1-753}$ transmembrane helices (TMs) as well as the LMNG molecule occupying the periplasmic vestibule, both colored as in Figure 1B.

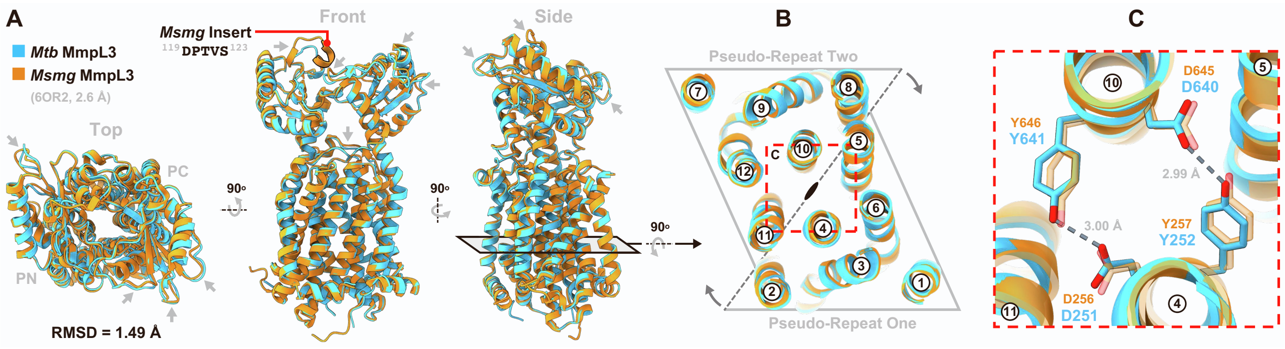

Figure S2. **Structural Comparisons of *Mtb* MmpL3$_{1\text{-}753}$ and *Msmg* MmpL3, Related to Figure 1**

(A) Alignment of the *Mtb* MmpL3$_{1\text{-}753}$ (blue) and *Msmg* MmpL3$_{1\text{-}773}$ (orange, PDB code: 6OR2, Su et al., 2019) models (1.49 Å RMSD over all atoms), shown in cartoon representation, and viewed from the membrane plane (front, side) or from the periplasm (top). Grey arrows emphasise deviations in loop architecture between the orthologs. A black outline, and accompanying annotation, identifies the PN pentapeptide (DPTVS) present in *Msmg* but not *Mtb* MmpL3. **(B)** Clipped view of the overlayed *Mtb* and *Msmg* MmpL3 transmembrane domains (TMDs), sliced as indicated in (A). The black oval designates the central 2-fold pseudo-symmetry axis, relating the two six transmembrane helix (TM) repeats (enclosed in grey triangles) that comprise the TMD. TMs are numbered. **(C)** Detailed close-up of region boxed in (B), showing the pair of Asp-Tyr dyads connecting TM4 and TM10 through hydrogen bonds (dashed lines) in the middle of the TMD. Colored as in (A), with side chains in stick form. Almost universally conserved in MmpLs, these residues are thought to be crucial for proton-motive force (PMF) transduction within the family. Notably, preclinical antituberculars targeting MmpL3 disrupt this Asp-Tyr hydrogen bond network; presumably de-energizing the transporter.

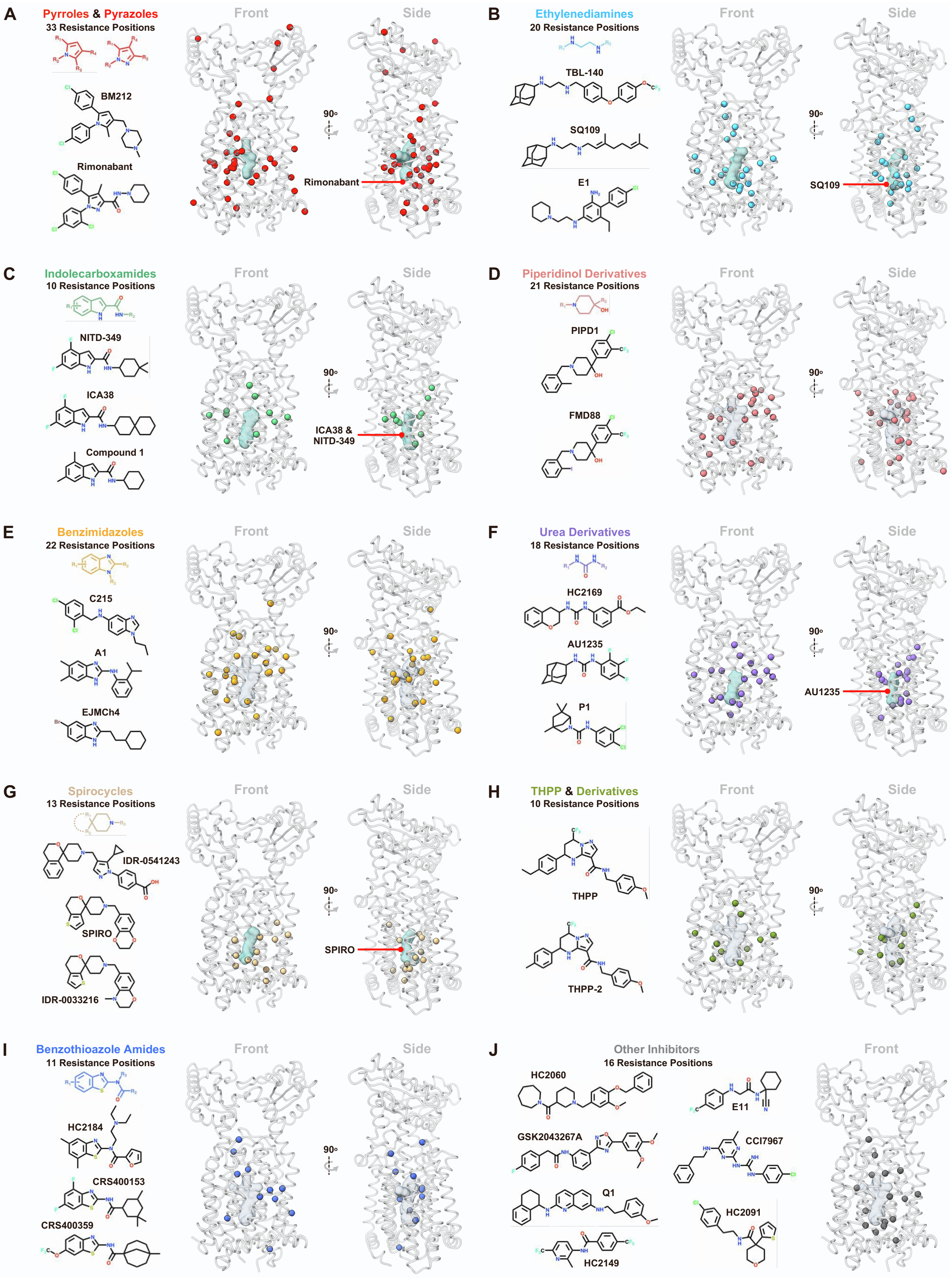

Figure S3. **Structural Mapping of MmpL3 Drug-Resistance Mutations Stratified by Inhibitor Class, Related to Figure 3 and Table S2**

(A – J) Discrete mapping, onto the *Mtb* $MmpL3_{1\text{-}753}$ structure, of collections of variants reported to confer resistance to members of each of the nine established classes of preclinical MmpL3 inhibitor (A – I), alongside a tenth miscellaneous grouping of predominantly orphan scaffolds (J). In each case resistance positions (RPs) are depicted by balls and the protein (transparent grey, licorice representation) viewed from one or more directions in the plane of the membrane. Where molecules of a given class have been co-crystallized with the *Msmg* homolog (PDB codes: 6AJG to 6AJJ, 7C2N & 7C2M, Zhang et al., 2019; Yang et al., 2020) a surface representation of the bound compound or compounds is superimposed (transparent teal) and annotated, otherwise the composite drug pocket is shown for reference (transparent grey, as in Figure 1C). Examples of agents affected by the plotted RPs are displayed for each family, and where informative a skeletal formula of the core class scaffold provided (R-groups denote variable substituents). Panels show RPs against (A) pyrrole/pyrazole, (B) ethylenediamine, (C) indolecarboxamide, (D) piperidinol derivative, (E) benzimidazole, (F) urea derivative, (G) spirocycle, (H) THPP associated, (I) benzothioazole amide, and (J) miscellaneous class MmpL3 inhibitors. All mutations are collated in Table S2.

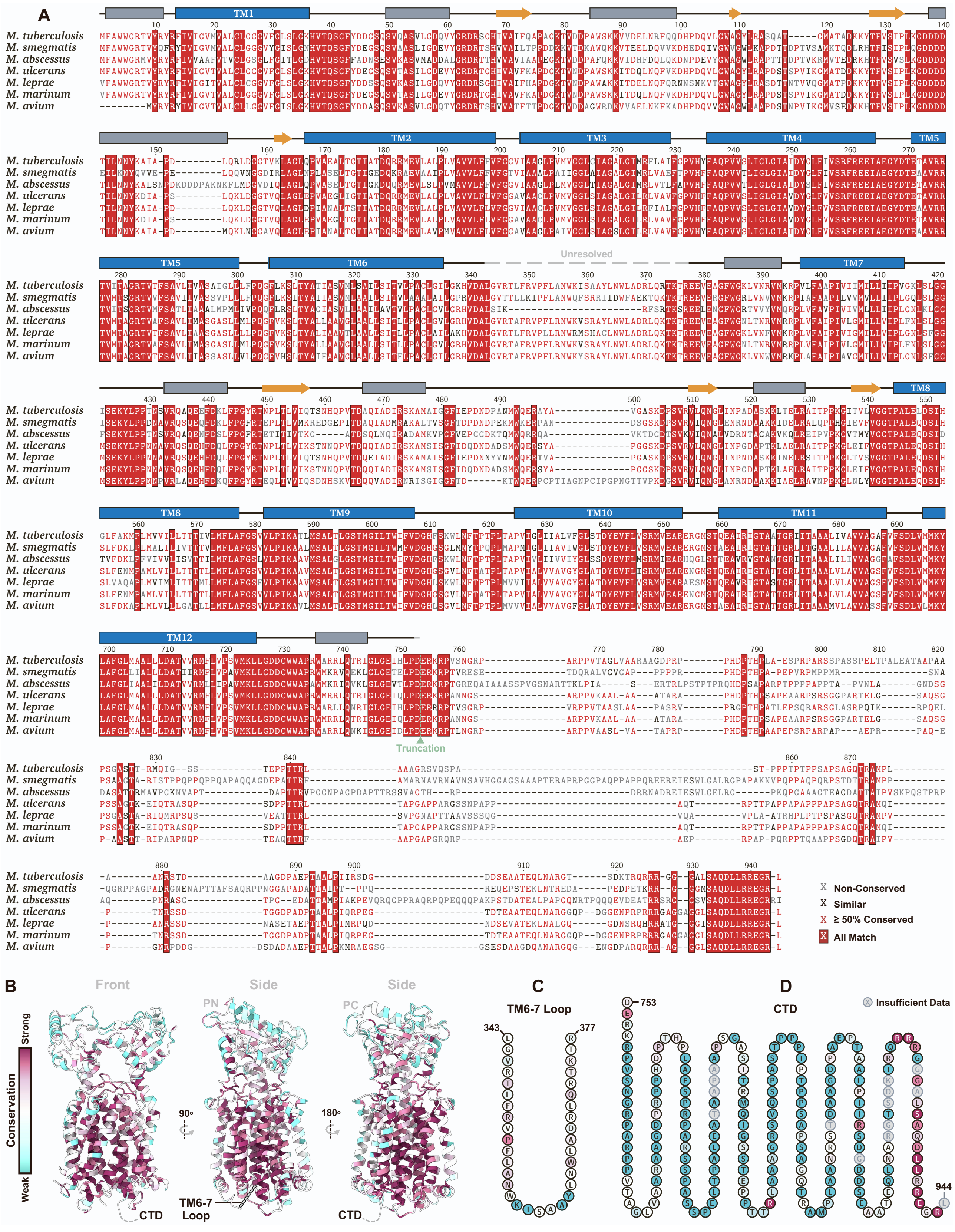

Figure S4. Ortholog Sequence Alignment and Conservation Analysis, Related to Figures 3 and 4

(A) Sequence alignment of *Mtb* MmpL3 with orthologs from *Msmg*, *M. abscessus*, *M. ulcerans*, *M. leprae*, *M. marinum*, and *M. avium*. Positions of secondary structure elements, derived from the *Mtb* MmpL3$_{1\text{-}753}$ model, are indicated — grey and blue rectangles denote non-transmembrane and transmembrane helices (TMs) respectively, and yellow arrows β-strands. Unresolved regions are shown by a dashed grey line and the C-terminal truncation at D753 is annotated. Residues conserved in all aligned orthologs are enclosed in red boxes. **(B)** Cartoon representation of *Mtb* MmpL3$_{1\text{-}753}$ colored by conservation, performed on the ConSurf server (Ashkenazy et al., 2016). Locations of the removed C-terminal domain (CTD) and unresolved TM6-7 loop are labelled. **(C)** & **(D)** ConSurf conservation of residues in the *Mtb* MmpL3 (C) TM6-7 loop (residues 343-377), and (D) CTD (residues 753-944); color-coded as in (B).

	Mtb MmpL3 (EMD-12604) (PDB: 7NVH)
**Data Collection & Processing**	
Magnification	105,000
Voltage (kV)	300
Electron Exposure (e-/Å^2)	58.2
Defocus Range (μm)	-3 to -0.5
Pixel Size (Å)	0.832
Symmetry Imposed	C1
Initial Particle Images (no.)	7,764,838
Final Particle Images (no.)	414,082
Map Resolution (Å)	3.0
FSC Threshold	0.143
Map Resolution Range (Å)	3.0 – 4.0
**Refinement**	
Initial Model Used (PDB Code)	6OR2
Model Resolution (Å)	3.0
FSC Threshold	0.143
Model Resolution Range (Å)	3.0 – 4.0
Map Sharpening *B* factor (Å^2)	-86.7
Model Composition	
Non-hydrogen Atoms	5529
Protein Residues	717
Ligands	1
B Factors (Å^2)	
Protein	53.19
Ligand	63.35
Root-mean-square Deviations	
Bond Lengths (Å)	0.004
Bond Angles (°)	0.641
Validation	
MolProbity Score	1.78
Clashscore	7.31
Poor Rotamers (%)	0.69
Ramachandran Plot	
Favoured (%)	94.39
Allowed (%)	5.61
Disallowed (%)	0

Table S1. **Cryo-EM Data Collection, Refinement and Model Validation Statistics, Related to Figure 1**
