# Supplementary material for: Cryo-EM structure and resistance landscape of M. tuberculosis MmpL3: An emergent therapeutic target
Source: Structure. 2021 Oct 7;29(10):1182–1191.e4. doi: 10.1016/j.str.2021.06.013 (PMC8752444; doi:10.1016/j.str.2021.06.013)
Supplement: Document S2. Article plus supplemental information [file mmc4.pdf]

Structure

Short Article

Cryo-EM structure and resistance landscape of *M. tuberculosis* MmpL3: An emergent therapeutic target

Graphical abstract

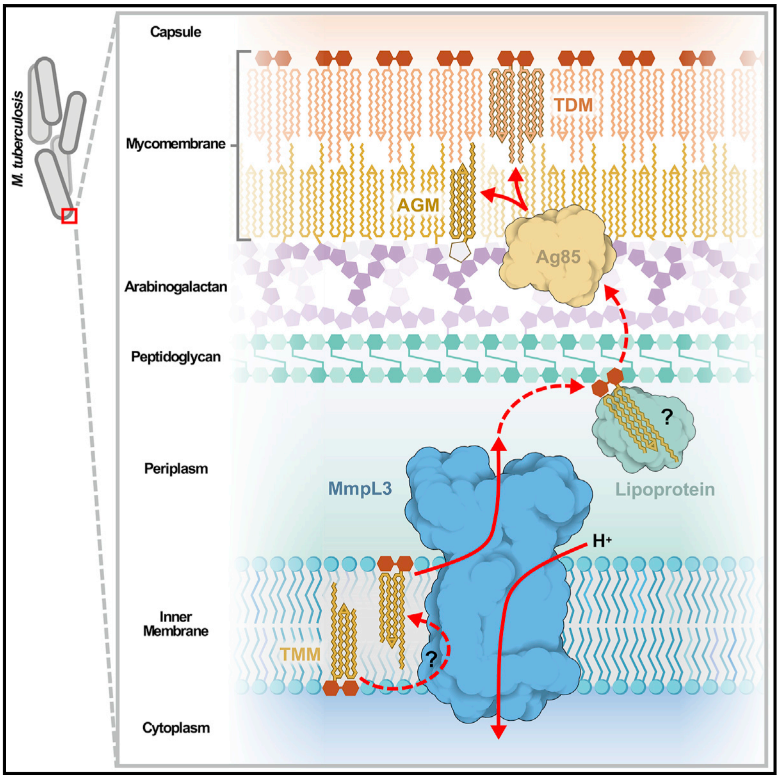

Highlights

- Cryo-EM structure of *M. tuberculosis* MmpL3 determined at 3.0 Å resolution
- An LMNG molecule within the periplasmic cavity suggests the TMM export pathway
- Comprehensive structural mapping of resistance-conferring MmpL3 variants
- Genome-mined MmpL3 mutations indicate minimal pre-existing resistance

Authors

Oliver Adams, Justin C. Deme, Joanne L. Parker, the CRyPTIC Consortium, Philip W. Fowler, Susan M. Lea, Simon Newstead

Correspondence

susan.lea@nih.gov (S.M.L.), simon.newstead@bioch.ox.ac.uk (S.N.)

In brief

Adams et al. report a structure of the *M. tuberculosis* glycolipid transporter MmpL3, a protein essential to envelope biogenesis and an emergent therapeutic target. The structure reveals the probable lipid export pathway, and mapping of resistance-conferring mutations suggests the existence of multiple routes to preclinical inhibitor evasion.

Adams et al., 2021, Structure *29*, 1182–1191
October 7, 2021 © 2021 The Author(s). Published by Elsevier Ltd.
https://doi.org/10.1016/j.str.2021.06.013

CellPress

CellPress
OPEN ACCESS

Structure

Short Article

Cryo-EM structure and resistance landscape of *M. tuberculosis* MmpL3: An emergent therapeutic target

Oliver Adams,[1] Justin C. Deme,[2,3,4] Joanne L. Parker,[1] the CRyPTIC Consortium,[8] Philip W. Fowler,[5,6] Susan M. Lea,[2,3,4,*] and Simon Newstead[1,7,9,*]
[1]Department of Biochemistry, University of Oxford, Oxford OX1 3QU, UK
[2]The Sir William Dunn School of Pathology, University of Oxford, Oxford OX1 3RE, UK
[3]Central Oxford Structural Molecular Imaging Centre (COSMIC), University of Oxford, Oxford OX1 3RE, UK
[4]Center for Structural Biology, Center for Cancer Research, National Cancer Institute, Frederick, MD 21702-1201, USA
[5]Nuffield Department of Medicine, John Radcliffe Hospital, University of Oxford, Oxford OX3 9DU, UK
[6]National Institute of Health Research (NIHR) Oxford Biomedical Research Centre, John Radcliffe, Oxford OX3 9DU, UK
[7]The Kavli Institute for Nanoscience Discovery, University of Oxford, Oxford OX1 3QU, UK
[8]A Corporate Author; for the List of Consortium Members see the section at the end of this article.
[9]Lead contact
*Correspondence: susan.lea@nih.gov (S.M.L.), simon.newstead@bioch.ox.ac.uk (S.N.)
https://doi.org/10.1016/j.str.2021.06.013

SUMMARY

Tuberculosis (TB) is the leading cause of death from a single infectious agent and in 2019 an estimated 10 million people worldwide contracted the disease. Although treatments for TB exist, continual emergence of drug-resistant variants necessitates urgent development of novel antituberculars. An important new target is the lipid transporter MmpL3, which is required for construction of the unique cell envelope that shields *Mycobacterium tuberculosis* (*Mtb*) from the immune system. However, a structural understanding of the mutations in *Mtb* MmpL3 that confer resistance to the many preclinical leads is lacking, hampering efforts to circumvent resistance mechanisms. Here, we present the cryoelectron microscopy structure of *Mtb* MmpL3 and use it to comprehensively analyze the mutational landscape of drug resistance. Our data provide a rational explanation for resistance variants local to the central drug binding site, and also highlight a potential alternative route to resistance operating within the periplasmic domain.

INTRODUCTION

Tuberculosis (TB) remains the leading cause of death attributable to a single pathogen (World Health Organization, 2020). Compounding this is the emergence and endemic nature of several drug-resistant *Mycobacterium tuberculosis* (*Mtb*) strains (Udwadia et al., 2012; Velayati et al., 2009). Mycobacterial membrane protein large 3 (MmpL3) is part of a vital biosynthetic pathway in *Mycobacterium* species and represents a promising new avenue for novel antitubercular drug development (Rayasam, 2014).

MmpL3 is an essential protein belonging to the resistance-nodulation-division (RND) superfamily of transporters (DeJesus et al., 2017; Domenech et al., 2005). Performing diverse functions throughout all domains of life, the RND superfamily were initially characterized as multi-drug efflux pumps in Gram-negative bacteria (Nikaido, 2018). In Gram-positive mycobacteria the MmpL family predominantly function as endogenous lipid transporters, with many, including MmpL3, involved in the biogenesis of the multi-layered cell envelope (Chalut, 2016; Viljoen et al., 2017). Specifically, MmpL3 exports intracellularly synthesized trehalose monomycolate (TMM) into the periplasm (Grzegorzewicz et al., 2012). TMM transport is essential for the formation of the unique *Mycobacterium* cell envelope that functions as the main physical barrier to drug entry and neutralization by the immune system (Dulberger et al., 2020; Jackson, 2014). The driving force for TMM export is thought to come from the proton-motive force (PMF) established across the inner membrane (Grzegorzewicz et al., 2012; Székely and Cole, 2016), although the precise mechanism by which the PMF drives lipid export remains unclear.

Disruption of envelope mycolation, as induced by small-molecule or genetic inhibition of MmpL3, has repeatedly been shown to be bactericidal (Degiacomi et al., 2017; Li et al., 2016; Shao et al., 2020; Varela et al., 2012). Indeed, the list of preclinical MmpL3 inhibitors is ever-growing, and features compounds with diverse chemical scaffolds (Dupont et al., 2016; De Groote et al., 2018; Grzegorzewicz et al., 2012; Lun et al., 2013; Remuiñán et al., 2013; La Rosa et al., 2012; Stanley et al., 2012; Tahlan et al., 2012). Encouragingly, some have been observed to synergize with existing antituberculars (Li et al., 2017; Nikonenko et al., 2007; Stec et al., 2016) and one, the ethylenediamine SQ109, has completed phase II clinical trials in various countries (Butler and Paterson, 2020; Degiacomi et al., 2020).

This is an open access article under the CC BY license (http://creativecommons.org/licenses/by/4.0/).

Significant insights into the interactions between MmpL3 and its inhibitors were recently reported using the *Mycobacterium smegmatis* (*Msmg*) ortholog (Yang et al., 2020; Zhang et al., 2019), which shares 61% sequence identity to *Mtb*. Nevertheless, given the severity of the TB pandemic, the evident susceptibility of MmpL3 to small-molecule inhibitors, and the numerous medicinal chemistry campaigns against it, understanding the structure and biochemical properties of the *Mtb* transporter remains a high priority.

Here, we report the cryoelectron microscopy (cryo-EM) structure of *Mtb* MmpL3 at a resolution of 3.0 Å. We present the most comprehensive structural mapping of MmpL3 variants to date, analyzing over 100 unique resistance-conferring substitutions selected by exposure to preclinical agents. In addition, we examine non-synonymous mutations mined from >45,000 whole-genome sequenced *Mtb* isolates. In conjunction with the recently reported structures of MmpL3 from *Msmg*, our work provides a foundation for structure-based drug design against *Mtb* MmpL3, guided by an enhanced appreciation of the underlying mutational landscape.

RESULTS AND DISCUSSION

Cryo-EM structure of *Mtb* MmpL3

To obtain the structure of *Mtb* MmpL3 we generated a C-terminally truncated (residues 1–753) construct capable of being overexpressed in *E. coli* (Figure S1A). The intracellular C-terminal domain (CTD) is dispensable for transport, with multiple C-terminal *Mtb* MmpL3 truncations viable *in vivo* (Belardinelli et al., 2016). Despite extensive efforts, $\text{MmpL3}_{1\text{-}753}$ proved recalcitrant to crystallization. However, cryo-EM imaging of the protein purified in lauryl maltose neopentyl glycol (LMNG) permitted its three-dimensional reconstruction to a resolution of 3.0 Å (Figures S1B–S1E). Our map reveals $\text{MmpL3}_{1\text{-}753}$ to be monomeric (Figure 1A), consistent with the *Msmg* homolog (Su et al., 2019; Zhang et al., 2019) (Figure S2A). The cryo-EM map was of sufficient quality to confidently model almost the entirety (residues 1–342, 378–752) of the construct (Figures 1B and S1F; Table S1). *Mtb* $\text{MmpL3}_{1\text{-}753}$ adopts a transmembrane domain (TMD) fold archetypal of RND proteins, consisting of 12 transmembrane helices (TMs) arranged as two sequence-contiguous bundles (TMs 1–6 and 7–12). These are related by a central 2-fold pseudo-symmetry axis running perpendicular to the membrane plane (Figure S2B). The $\text{MmpL3}_{1\text{-}753}$ TMD is braced against the inner leaflet of the cytoplasmic membrane via three short, lateral, amphipathic α helices, two preceding TMs 1 and 7, with the third leading into the CTD. Characteristic of RND transporters, sizable periplasmic loops connect TMs 1–2 in the N-terminal half of $\text{MmpL3}_{1\text{-}753}$ (PN, residues 37–166) and TMs 7–8 (PC, residues 415–544) in the C-terminal half of the molecule. Both display an α-β-α-β-α-β topology, with the first α helix of each contributing to the tertiary structure of the adjacent loop. This partial interdigitation of secondary structural elements results in PN and PC clasping one another to give a singular periplasmic domain (PD), capping the TMD and sharing its pseudo-symmetry axis. The N termini of TM2 and TM8 are longer than the other helices and extend into the PD (Figure 1C), which itself protrudes over 40 Å into the periplasmic space.

Similar to structures of the *Msmg* MmpL3 homolog (Su et al., 2019; Zhang et al., 2019), the *Mtb* protein has an extensive cavity enclosed by the PD (Figure 1C). The cavity comprises a central vestibule between the PN and PC lobes, out of which branch two descending channels, each constricted in the vicinity of the PD-TMD "neck." On opposing faces of the protein these channels exit into the outer leaflet of the inner membrane via grooves bounded by TMs 1–4 or 7–10, respectively. The upper PD chamber likewise possesses three apertures opening into the periplasm from the front (PD_F), back (PD_B), and top (PD_T) of the molecule and each gated by a combination of charged and hydroxyl-containing side chains (Figure 1D). Although the role of this cavity has not yet been fully elucidated, its position within the molecule and relationship to the outer leaflet suggests it forms the conduit through which TMM is extracted into the periplasm, a role supported by site-directed mutagenesis (Belardinelli et al., 2016). Within this cavity a lipid-like species was captured in our structure, most likely the detergent LMNG, which adopts a splayed arrangement bound between the PN and PC subdomains (Figures 1A, 1B, and 2A). LMNG creates a substantial interface, packing against 35 residues (within 4.5 Å) and sitting within the suggested transport path for TMM (Klenotic et al., 2020). The interactions are predominantly contributed by PN, PC, and TM2, but also feature side chains at the N terminus of TM8 and in the loops connecting TMs 3–4 and 11–12 (Figure 2A). Although the LMNG tails point in divergent directions, one halfway down into the channel exiting via TMs 1–4 and the other up toward PD_T, both are accommodated in pockets partially scaffolded by TM2 aliphatic residues (L166, V169, L173, and I177), and end in aromatic residues (Y235 and F236 or F440 and Y447) provided by nearby secondary structure elements. In contrast, the two LMNG maltose head groups bifurcate to be individually coordinated by residues of the PD_F and PD_B apertures. In doing so their hydroxyls are hydrogen bonded to side chains of D58 and D139, as well as the backbone carbonyl of I422, leaving the terminal saccharide of each head group to be partly solvent exposed as it emerges through its respective PD opening.

A key question in the mechanism of MmpL3 is how this protein handles the large and complex TMM lipid. The previous *Msmg* MmpL3 structures were co-crystallized with three unique PD occupants (Figure 2B); pairs of the detergents lauryl-6-trehaloside (6DDTre, a structural analog of TMM) and dodecyl maltoside (DDM) (Zhang et al., 2019), as well as a single phosphatidylethanolamine (PE) lipid (Su et al., 2019). Structural comparison with the *Mtb* $\text{MmpL3}_{1\text{-}753}$-LMNG complex identifies striking similarities in the binding poses of all four ligands (Figure 2C). The pockets embracing the LMNG alkyl chains in our structure are also exploited by 6DDTre and DDM to accommodate their respective hydrophobic tails. PE, in contrast, sits with its shorter heptadecanoate acyl chain in the upper of these two tail sites, leaving its peripheral carbons to extend through PD_T. The comparison of *Mtb* MmpL3 and *Msmg* MmpL3 suggests a "division of labor" model for PD lipid handling, wherein the central vestibule sequesters the alkyl chains away from the bulk periplasm, potentially into specific pockets (Figure 2C), while the proximate hydrophilic openings (PD_F, PD_B, and PD_T) bind the polar head group, coordinating it through hydrogen bonds and providing it a degree of solvent access. Segmentation of binding would permit accommodation of lipidic cargo in, and subsequent

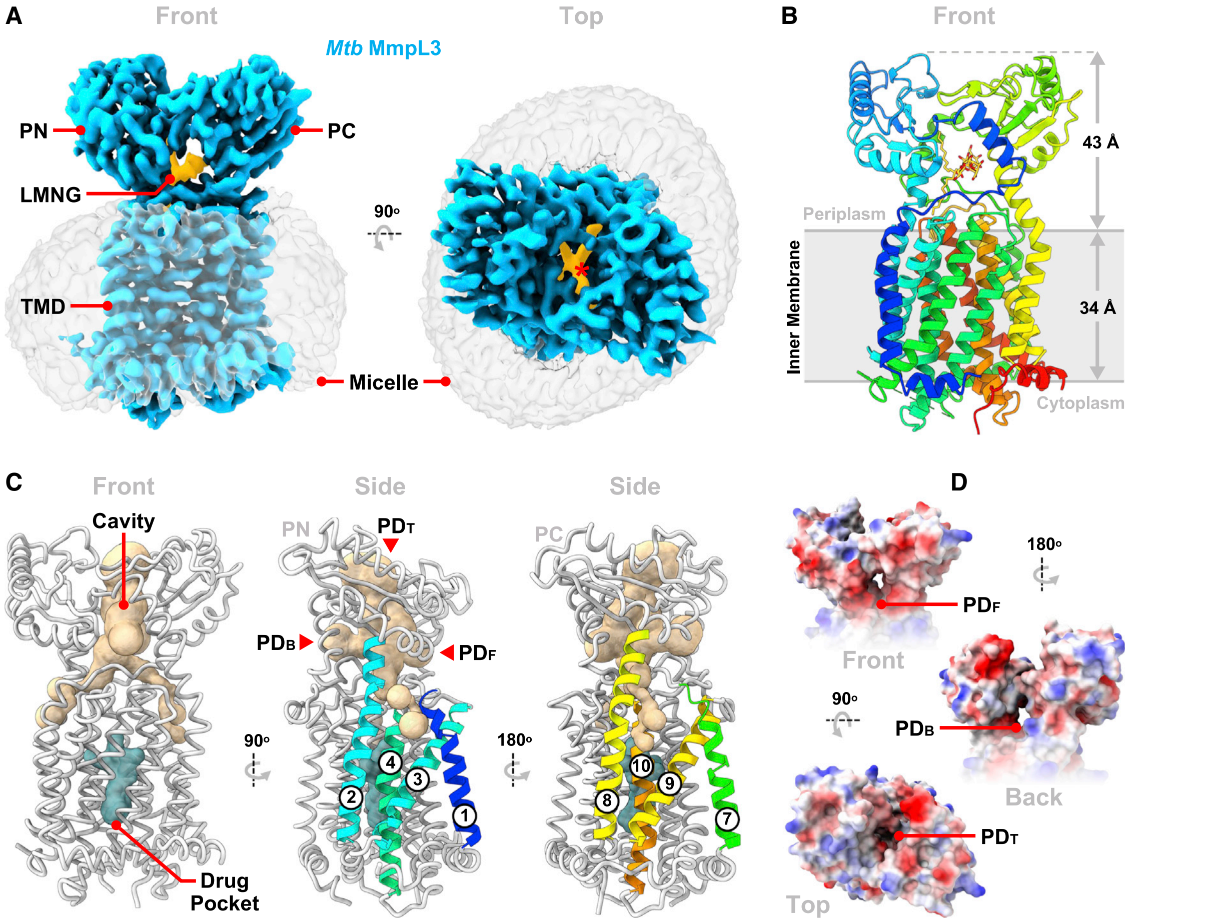

Figure 1. Cryo-EM map and structural analysis of *Mtb* MmpL3

(A) Cryo-EM density of *Mtb* $MmpL3_{1\text{-}753}$ as viewed from the membrane plane (front), or perpendicular to it from the periplasm (top). Proteinaceous density is blue (contour level 0.427, sharpened map), the surrounding detergent micelle gray, and a bound LMNG molecule gold (contour level 0.150, unsharpened map). Annotations highlight the transmembrane domain (TMD), and the N-terminal (PN) and C-terminal (PC) lobes of the periplasmic domain (PD).

(B) Cartoon representation of the *Mtb* $MmpL3_{1\text{-}753}$ atomic model, colored blue (N terminus) to red (C terminus). LMNG is shown as sticks (gold).

(C) Licorice representations of *Mtb* $MmpL3_{1\text{-}753}$ (gray) accompanied by surface depictions emphasizing its PD cavity network (wheat, calculated using Caver Analyst 2.0 [Jurcik et al., 2018]) and TMD drug binding pocket (teal). The TMs (1–4 and 7–10) lining cavity exits into the outer leaflet are indicated.

(D) Electrostatic surface renderings of the $MmpL3_{1\text{-}753}$ PD, highlighting the hydrophilic residues gating the labeled PD_F, PD_B, and PD_T cavity openings.

See also Figures S1 and S2, and Table S1.

manipulation into, diverse conformations. This flexibility is plausibly essential for translocation of substrates as large as TMM (molecular weight >1.4 kDa, Fujita et al., 2005), and likely underlies the lipid binding promiscuity observed for *Msmg* MmpL3 by native mass spectrometry (Su et al., 2019).

Mapping MmpL3 mutations

MmpL3 is the target of many preclinical antituberculars (Degiacomi et al., 2020). These compounds target a conserved central pocket within the TMD that sits at the interface between TMs 4 and 5 and 10 and 11 (Figures 1C and S4B) and incorporates the Asp-Tyr interaction network (Figure S2C) proposed to function in coupling TMM transport to the PMF (Bernut et al., 2016; Yang et al., 2020; Zhang et al., 2019). Antitubercular agents have been predominantly discovered by high-throughput whole-cell phenotypic screening against sizable chemical libraries; with targets for active compounds inferred by their mutation in whole-genome-sequenced resistant isolates (Goldman, 2013). Consequently, there exists a substantial repository of drug-resistant MmpL3 variants in the literature, which we collated and mapped onto our $MmpL3_{1\text{-}753}$ model. We aggregated 112 unique resistance-conferring mutations occurring at 83 different positions in MmpL3, 21 of which differ between the *Msmg* and *Mtb* homologs (Table S2). Of the 112 mutations, 109 variants at 80 positions were mappable onto our *Mtb* $MmpL3_{1\text{-}753}$ structure (Figure 3A). The remaining positions populated the unresolved inter-repeat linker. Most mutations were described in *Mtb* (57 positions), but to provide a more complete picture of the resistance landscape we also back-mapped variants originally identified in *Msmg* (11 positions), *M. bovis* (13 positions), and *M. abscessus* (19 positions) by sequence alignment (Figure S4A).

Of the 83 resistance positions (RPs) identified, ~90% reside in the TMD and 60% are within 10 Å of the drug pocket (Figure 3A). Such clustering suggests that the dominant route to resistance is

CellPress
OPEN ACCESS

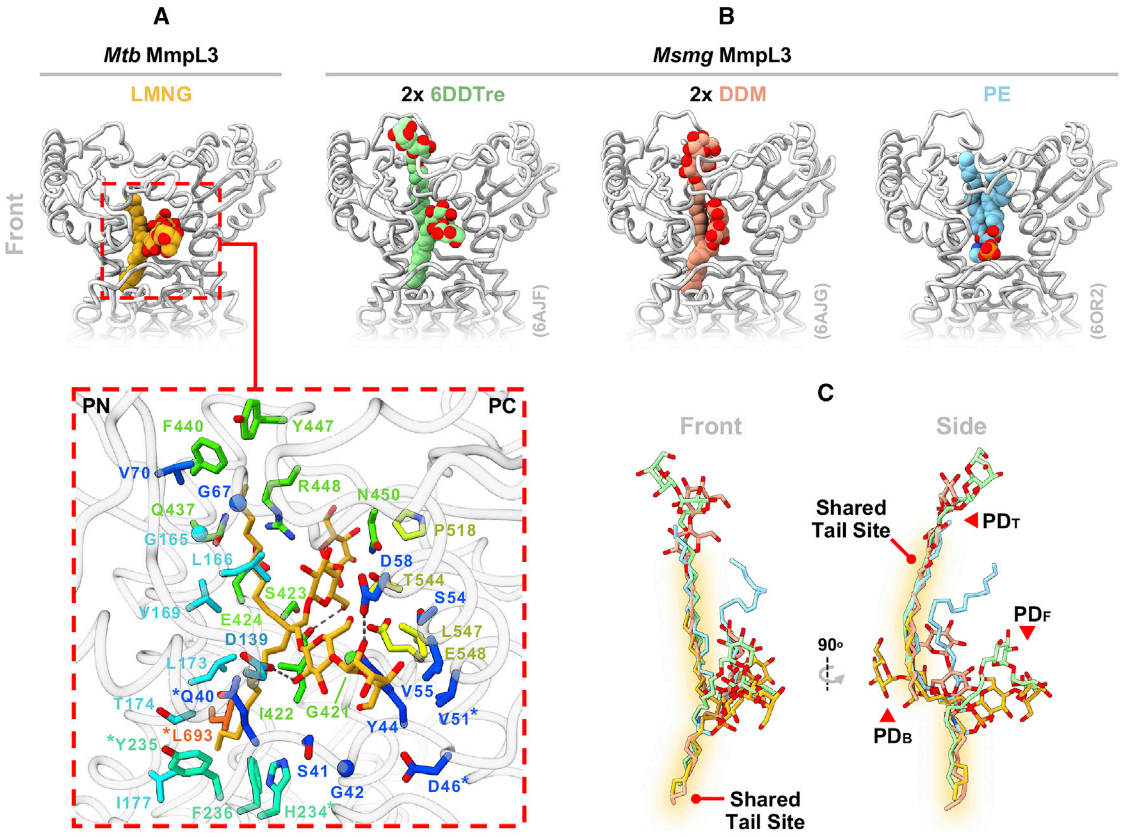

Figure 2. Comparison of MmpL3 periplasmic ligands

(A) Close-up of the *Mtb* $MmpL3_{1-753}$ PD LMNG. Residues within 4.5 Å, plus the detergent itself (gold), are shown as sticks; bar glycines shown as balls. Side chains are rainbow colored, as in Figure 1B. Dashed lines denote hydrogen bonds. Asterisks highlight residues conferring inhibitor resistance when mutated.

(B) Side-by-side comparison of the 6DDTre (green), DDM (salmon), and PE (blue) molecules co-crystallized within the *Msmg* MmpL3 PD. Ligands are shown as spheres and proteins (gray) in licorice representation. PDB codes are in brackets.

(C) Overlay of the *Mtb* and *Msmg* PD ligands, each depicted as sticks and colored as in (A) and (B). Common alkyl chain sites, and the positions of the PD apertures, are annotated.

through mutation of residues defining the 2-fold pseudo-symmetry axis relating the two six-helix bundles of the TMD. These mutations likely weaken the affinity of antitubercular drugs targeting the transmembrane site, as has been demonstrated for a handful of *Msmg* MmpL3 variants by MST (Zhang et al., 2019). This conclusion is also consistent with proposals that the magnitude and spectrum of the resistance phenotype given by an MmpL3 mutant correlates with its proximity to this pocket (Williams et al., 2019). However, our analysis reveals that a significant proportion of RPs are located >10 Å away from the drug pocket, including some within the LMNG binding site (asterisks in Figure 2A), suggesting that there is at least one additional, and as yet uncharacterized, MmpL3 resistance mechanism; likely involving the PD and its cavity. Future studies exploring the role of the PD within the mechanism of MmpL3 will be required to uncover how these mutations circumvent the action of drugs targeting the TMD.

To further dissect the resistance landscape, we categorized the RPs by the nine established MmpL3 inhibitor classes, as well as a tenth miscellaneous grouping of orphan scaffolds (Figure S3). All the scaffolds retained RP clustering around the common drug binding pocket, corroborating its use by classes yet to be structurally characterized in complex with MmpL3. Armed with our RP mapping data we undertook a finer analysis with respect to the drug binding poses resolved in the *Msmg* structures. This highlighted that class-specific differences in RP distribution can be attributed to subtleties in the interactions each makes with MmpL3. The most obvious example are the pyrrole/pyrazole class inhibitors, where RPs are enriched around a subsite (residues L243, V681, V684, A685, and L703) unused in the binding of other structurally characterized scaffolds (Figure S3A) (Zhang et al., 2019). Equally, the differences apparent between indolecarboxamides (Figure S3C) and spirocycles (Figure S3G) agrees with observations that SPIRO, an example of the latter, makes fewer interactions at the top of the shared inhibitor pocket, but more at the bottom, relative to NITD-349, a member of the former family (Yang et al., 2020). Thus, despite significant overlap, there is clear differentiation in the pattern of RPs selected by each drug class.

A greater understanding of the MmpL3 mutations capable of conferring resistance to multiple scaffolds is critical to design of agents less easily inducing resistance. Such knowledge, for example, could direct medicinal chemistry efforts toward minimizing lead interactions with the offending residues. We

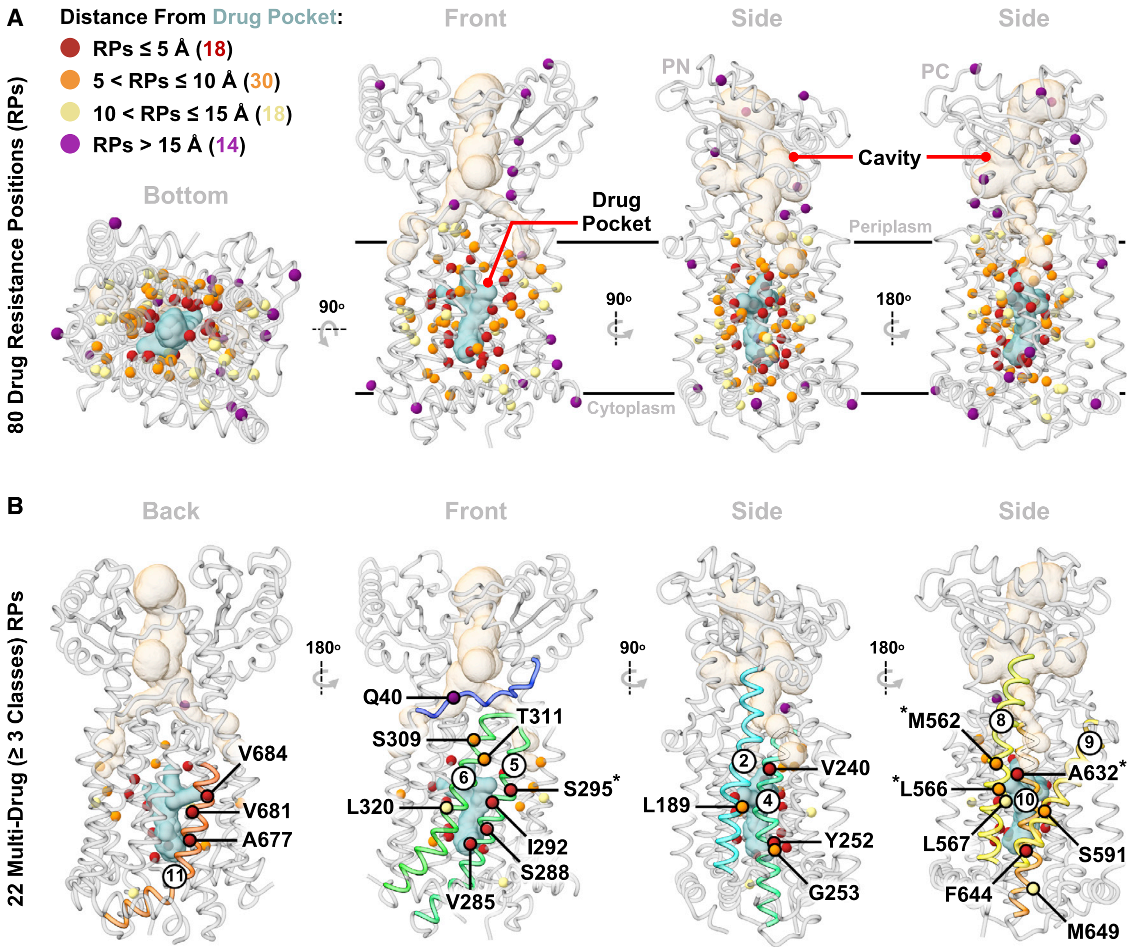

Figure 3. Mapping MmpL3 drug-resistance mutations

(A) Eighty resistance positions (RPs) plotted onto *Mtb* MmpL3$_{1\text{-}753}$ as balls, color-coded by distance from the universal drug pocket (teal). The PD cavity is also shown (wheat surface).

(B) Residues of the 22 RPs causing cross-resistance to a minimum of three classes of MmpL3 inhibitor. Black asterisks denote cross-RPs only observed in *M. abscessus* MmpL3.

See also Figures S3 and S4, and Table S2.

therefore filtered for RPs where variants have been associated with resistance to at least three MmpL3 inhibitor classes (Figure 3B). Twenty-two RPs were identified, all except Q40 fell within the TMD and 50% were within 5 Å of the common binding pocket. Mutations of Q40, which resides in the linker joining TM1 to the first PN helix and is >15 Å from the drug binding site, almost certainly act by the aforementioned "alternative" resistance mechanism. Consistently, the Q40R variant failed to alter *Msmg* MmpL3 affinity for inhibitors as measured by MST (Zhang et al., 2019). Q40 lines the MmpL3$_{1\text{-}753}$ channel exiting via TMs 1–4, within the LMNG binding site, and has thus far only been reported to confer resistance phenotypes on mutation to cationic amino acids (R or H, Table S2). It represents an important site for future biochemical studies.

Structural interpretation of the physiochemical changes introduced by variants at the other 21 cross-RPs identifies a number of plausible explanations for their phenotypes. A facile route to resistance, with minimal fold disruption, would be conservative binding site mutations that alter side chain size, thereby impairing steric complementarity. This mechanism is apparent for three *Mtb* drug-interacting valines (V240, V285, and V684) that mutate to smaller residues (A or G, Table S2) to achieve multi-class resistance. The reverse mechanism, enlarging side chains to promote steric clashes with complexed inhibitors, is employed at two drug-interacting sites, where alanine side chains (A632 and A677) are substituted for bulkier aliphatics or aromatics (Table S2). Modulation of residue hydrogen bond capacity is another route to resistance, either through loss (T311I, S591I), gain (I292S/T, L566S), or exchange (S288T) of hydroxyl-containing side chains (Table S2). Further away from the shared drug pocket many of the substitutions at cross-RPs result in dramatic chemical perturbations, in line with both their presumed action at a distance, and a diminished capacity to collaterally incapacitate the proton-relay system. These less-conservative variants either introduce additional charge into the TMD (L189R and G253E) or supply intra-TM prolines (S295P, L320P, and L567P, Table S2), which is conformationally disruptive. Interestingly, four of the 22 cross-RPs have so far only been observed in *M. abscessus*

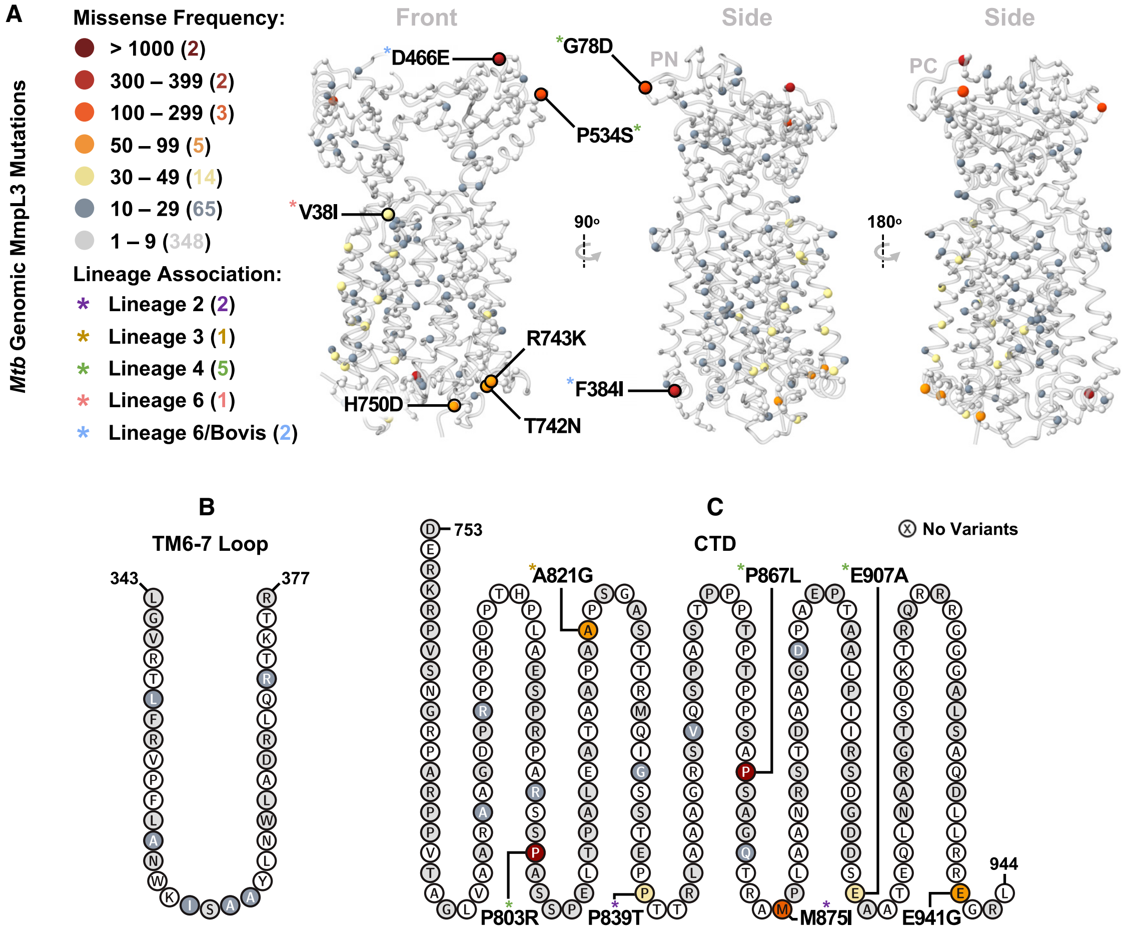

Figure 4. Wider non-synonymous landscape of MmpL3

(A) Structural mapping of MmpL3 variants mined from over 45,000 *Mtb* genomes, represented by balls and color-coded by mutational frequency. The most abundant substitutions, and/or those with apparent *Mtb* lineage association (colored asterisks) are labeled.

(B and C) (B) Incidence of non-synonymous mutations in the TM6-7 loop, and (C) C-terminal domain.

See also Figure S4 and Table S3.

(asterisks in Figure 3B). This suggests that MmpL3 orthologs may preferentially mutate at different residues to facilitate drug escape, an important caveat if future antitubercular MmpL3 inhibitors are to be repurposed for treatment of non-tuberculous mycobacterial infections (Li et al., 2018).

A large number of *Mtb* genomes (45,878), mostly originating from clinical samples, are available from the European Nucleotide Archive (ENA). By analyzing these with respect to our resistance catalog (Table S2) one can estimate how much environmental resistance any new antibiotics targeting MmpL3 are likely to encounter. Consistent with its essential nature, no MmpL3 mutations were detected in the majority of genomes; 3,890 (8.5%) out of the 45,878 samples harbored 7,475 non-synonymous mutations (Table S3). In total, 597 distinct amino acid substitutions were identified; however, the majority (422, 71%) were seen three or fewer times. Only 25 mutations were detected in 30 or more samples and accounted for 5,374 (72%) of the non-synonymous changes.

These 25 mutations are found throughout the protein (Figure 4) and none were identified by the *in vitro* resistance studies (Table S2). While we cannot conclude that they do not confer resistance, one can infer that it is unlikely. As may be expected, the chemical perturbation introduced by these variants tends to be smaller in the TMD than in the CTD, mirroring domain conservation (Figures S4B–S4D). A subset also appear specific to particular *Mtb* lineages (asterisks in Figure 4), suggesting that they are ancestral (Comas et al., 2013). Conversely, the lack of abundant non-lineage defining mutations (Table S3) implies there is currently little evolutionary pressure on the *mmpL3* gene and/or non-synonymous mutations tend to be deleterious.

The most prevalent resistance-conferring mutation in the genome-mined dataset, V210A, occurs in just 21 samples. Although 16 of these are found in but three studies (Colangeli et al., 2018; Guerra-Assunçaõ et al., 2015; Pankhurst et al., 2016), all three studies sequence clinical samples that can reasonably be expected not to have been exposed to any of the novel MmpL3 inhibitors under development. A further 12 mutations identified by experiment as conferring resistance are present in an additional 32 samples in the ENA (Table S3), but only three (G628A, Y252C, and I585V) are found in more than five

isolates. Simply counting these samples, and bearing in mind that the *Mtb* genomes deposited in the ENA are likely to be enriched for resistance, we arrive at 0.1% as an upper limit for the prevalence of pre-existing resistance mutations in MmpL3. This low percentage provides evidence that MmpL3 inhibitors, once licensed, are unlikely to encounter resistant variants in the environment.

In summary, our results provide a structural model of *Mtb* MmpL3, which, combined with our genetic analyses, provides a robust framework for antitubercular development against this essential protein.

MEMBERS OF THE CRyPTIC CONSORTIUM

Derrick W. Crook, Timothy E.A. Peto, A. Sarah Walker, Sarah J. Hoosdally, Ana L. Gibertoni Cruz, Joshua Carter, Alice Brankin, Sarah Earle, Samaneh Kouchaki, Alexander S. Lachapelle, Yang Yang, Timothy M. Walker, Philip W. Fowler, Daniel Wilson, and David A. Clifton, University of Oxford; Zamin Iqbal, Martin Hunt, Kerri M. Malone, Penelope Wintringer, Brice Letcher, and Jeff Knaggs, European Bioinformatics Institute; Daniela M. Cirillo, Emanuele Borroni, Simone Battaglia, Arash Ghodousi, Andrea Spitaleri, and Andrea Cabibbe, Emerging Bacterial Pathogens Unit, IRCCS San Raffaele Scientific Institute, Milan; Sabira Tahseen, National Tuberculosis Control Program Pakistan, Islamabad; Kayzad Nilgiriwala and Sanchi Shah, The Foundation for Medical Research, Mumbai; Camilla Rodrigues, Priti Kambli, Utkarsha Surve, and Rukhsar Khot, P.D. Hinduja National Hospital and Medical Research Center, Mumbai; Stefan Niemann, Thomas Kohl, and Matthias Merker, Research Center Borstel; Harald Hoffmann, Katharina Todt, and Sara Plesnik, Institute of Microbiology and Laboratory Medicine, IML red, Gauting; Nazir Ismail, Shaheed Vally Omar, Lavania Joseph Dumisani Ngcamu, Nana Okozi, and Shen Yuan Yao, National Institute for Communicable Diseases, Johannesburg; Guy Thwaites, Thuong Nguyen Thuy Thuong, Nhung Hoang Ngoc, and Vijay Srinivasan, Oxford University Clinical Research Unit, Ho Chi Minh City; David Moore, Jorge Coronel, and Walter Solano, London School of Hygiene and Tropical Medicine and Universidad Peruana Cayetano Heredia, Lima; George F. Gao, Guangxue He, Yanlin Zhao, Aijing Ma, and Chunfa Liu, China CDC, Beijing; Baoli Zhu, Institute of Microbiology, CAS, Beijing; Ian Laurenson and Pauline Claxton, Scottish Mycobacteria Reference Laboratory, Edinburgh; Robert J. Wilkinson, University of Cape Town, Imperial College London, and Francis Crick Institute; Anastasia Koch, University of Cape Town; Ajit Lalvani, Imperial College London; James Posey, CDC Atlanta; Jennifer Gardy, University of British Columbia; Jim Werngren, Public Health Agency of Sweden; Nicholas Paton, National University of Singapore; Ruwen Jou, Mei-Hua Wu, and Yu-Xin Xiao, CDC Taiwan; Lucilaine Ferrazoli, Rosangela Siqueira de Oliveira, and Juliana Maira Watanabe Pinhata, Institute Adolfo Lutz, São Paulo; James Millard, Africa Health Research Institute, Durban; Rob Warren, University of Stellenbosch, Cape Town; Annelies Van Rie, University of Antwerp; Simon Grandjean Lapierre, Marie-Sylvianne Rabodoarivelo, and Niaina Rakotosamimanana, Institut Pasteur de Madagascar; Camus Nimmo, University College London; Kimberlee Musser and Vincent Escuyer, Wadsworth Center, New York; Ted Cohen, Yale University; E. Grace Smith, Priti Rathod, Lisa Jarrett, and Daniela Matias, Public Health England, Birmingham.

SUPPORTING CITATIONS

The following references appear in the supplemental information: Dupont et al., 2019; Foss et al., 2016; Grover et al., 2021; Ioerger et al., 2013; Korycka-Machała et al., 2019; Kozikowski et al., 2017; Li et al., 2019; Li et al., 2020; McNeil et al., 2017; McNeil et al., 2020; Molin et al., 2019; Pandya et al., 2019; Poce et al., 2013; Poce et al., 2019; Rao et al., 2013; Raynaud et al., 2020; Shetty et al., 2018; Tantry et al., 2015; Trofimov et al., 2018; Zheng et al., 2018.

STAR★METHODS

Detailed methods are provided in the online version of this paper and include the following:

- KEY RESOURCES TABLE
- RESOURCE AVAILABILITY
  - Lead contact
  - Materials availability
  - Data and code availability
- EXPERIMENTAL MODEL AND SUBJECT DETAILS
- METHOD DETAILS
  - *Mtb* $MmpL3_{1\text{-}753}$ molecular cloning & purification
  - Cryo-EM sample preparation & imaging
  - Cryo-EM data processing
  - Model building & refinement
  - Structural analysis & data Visualisation
  - *Mtb* MmpL3 mutation collation
- QUANTIFICATION AND STATISTICAL ANALYSIS

SUPPLEMENTAL INFORMATION

Supplemental information can be found online at https://doi.org/10.1016/j.str.2021.06.013.

ACKNOWLEDGMENTS

This work was supported by a Biotechnology and Biological Sciences Research Council (UKRI-BBSRC) studentship to O.A. (BB/M011224/1), Wellcome awards to S.M.L. (209194; 100298) and S.N. (215519; 219531), and through MRC grants to S.M.L. (MR/M011984/1) and J.L.P. (MR/S021043/1). Research in P.W.F.'s laboratory is supported by the NIHR Oxford Biomedical Research Center, Oxford University Hospitals NHS Foundation Trust, John Radcliffe Hospital, Oxford, UK, a Wellcome Enriching Engagement grant and Computational Biomedicine, an EU Horizon 2020 Center of Excellence (823712). We acknowledge use of the Central Oxford Structural Molecular Imaging Center (COSMIC), which is supported by Wellcome award (201536), the EPA Cephalosporin Trust, and a Royal Society/Wolfson Foundation Laboratory Refurbishment grant (WL160052). The CRyPTIC Consortium was supported by grants from the Bill and Melinda Gates Foundation (OPP1133541) and a Wellcome /Newton Fund-MRC Collaborative Award (200205/Z/15/Z). The views expressed are those of the authors and not necessarily those of the NHS, the NIHR, or the Department of Health.

AUTHOR CONTRIBUTIONS

S.N., J.L.P., and P.W.F. conceived the project. O.A. performed all cloning and protein preparation. J.C.D. and S.M.L. performed all cryo-EM sample processing, data collection, and image analysis. O.A. and J.C.D. constructed the

atomic model. O.A. and P.W.F. conducted all bioinformatic and sequence-based analysis. O.A., P.W.F., J.L.P., and S.N. wrote the manuscript and prepared figures with contributions from J.C.D. and S.M.L.

DECLARATIONS OF INTERESTS

The authors declare no competing interests.

Received: April 12, 2021
Revised: May 24, 2021
Accepted: June 14, 2021
Published: November 4, 2021

REFERENCES

Asarnow, D., Palovcak, E., and Cheng, Y. (2019). asarnow/pyem: UCSF pyem v0.5. http://doi.org/10.5281/zenodo.3576630.

Ashkenazy, H., Abadi, S., Martz, E., Chay, O., Mayrose, I., Pupko, T., and Ben-Tal, N. (2016). ConSurf 2016: an improved methodology to estimate and visualize evolutionary conservation in macromolecules. Nucleic Acids Res. *44*, W344–W350.

Beitz, E. (2000a). TEXshade: shading and labeling of multiple sequence alignments using LATEX 2ε. Bioinformatics *16*, 135–139.

Beitz, E. (2000b). TEXtopo: shaded membrane protein topology plots in LATEX 2ε. Bioinformatics *16*, 1050–1051.

Belardinelli, J.M., Yazidi, A., Yang, L., Fabre, L., Li, W., Jacques, B., Angala, S.K., Rouiller, I., Zgurskaya, H.I., Sygusch, J., et al. (2016). Structure-function profile of MmpL3, the essential mycolic acid transporter from *Mycobacterium tuberculosis*. ACS Infect. Dis. *2*, 702–713.

Bernut, A., Viljoen, A., Dupont, C., Sapriel, G., Blaise, M., Bouchier, C., Brosch, R., de Chastellier, C., Herrmann, J.L., and Kremer, L. (2016). Insights into the smooth-to-rough transitioning in *Mycobacterium bolletii* unravels a functional Tyr residue conserved in all mycobacterial MmpL family members. Mol. Microbiol. *99*, 866–883.

Butler, M.S., and Paterson, D.L. (2020). Antibiotics in the clinical pipeline in October 2019. J. Antibiot. *73*, 329–364.

Caesar, J., Reboul, C.F., Machello, C., Kiesewetter, S., Tang, M.L., Deme, J.C., Johnson, S., Elmlund, D., Lea, S.M., and Elmlund, H. (2020). SIMPLE 3.0. Stream single-particle cryo-EM analysis in real time. J. Struct. Biol. X *4*, 100040.

Chalut, C. (2016). MmpL transporter-mediated export of cell-wall associated lipids and siderophores in mycobacteria. Tuberculosis *100*, 32–45.

Colangeli, R., Jedrey, H., Kim, S., Connell, R., Ma, S., Chippada Venkata, U.D., Chakravorty, S., Gupta, A., Sizemore, E.E., Diem, L., et al. (2018). Bacterial factors that predict relapse after tuberculosis therapy. New Engl. J. Med. *379*, 823–833.

Comas, I., Coscolla, M., Luo, T., Borrell, S., Holt, K.E., Kato-Maeda, M., Parkhill, J., Malla, B., Berg, S., Thwaites, G., et al. (2013). Out-of-Africa migration and Neolithic coexpansion of *Mycobacterium tuberculosis* with modern humans. Nat. Genet. *45*, 1176–1182.

Degiacomi, G., Benjak, A., Madacki, J., Boldrin, F., Provvedi, R., Palù, G., Kordulakova, J., Cole, S.T., and Manganelli, R. (2017). Essentiality of *mmpL3* and impact of its silencing on *Mycobacterium tuberculosis* gene expression. Sci. Rep. *7*, 43495.

Degiacomi, G., Belardinelli, J.M., Pasca, M.R., de Rossi, E., Riccardi, G., and Chiarelli, L.R. (2020). Promiscuous targets for antitubercular drug discovery: the paradigm of DprE1 and MmpL3. Appl. Sci. *10*, 623.

DeJesus, M.A., Gerrick, E.R., Xu, W., Park, S.W., Long, J.E., Boutte, C.C., Rubin, E.J., Schnappinger, D., Ehrt, S., Fortune, S.M., et al. (2017). Comprehensive essentiality analysis of the *Mycobacterium tuberculosis* genome via saturating transposon mutagenesis. mBio *8*, 1–17.

Domenech, P., Reed, M.B., and Barry, C.E. (2005). Contribution of the *Mycobacterium tuberculosis* MmpL protein family to virulence and drug resistance. Infect. Immun. *73*, 3492–3501.

Drew, D.E., Heijne, G.V., Nordlund, P., and de Gier, J.L. (2001). Green fluorescent protein as an indicator to monitor membrane protein overexpression in *Escherichia coli*. FEBS Lett. *507*, 220–224.

Drew, D., Slotboom, D.-J., Friso, G., Reda, T., Genevaux, P., Rapp, M., Meindl-Beinker, N.M., Lambert, W., Lerch, M., Daley, D.O., et al. (2005). A scalable, GFP-based pipeline for membrane protein overexpression screening and purification. Protein Sci. *14*, 2011–2017.

Dulberger, C.L., Rubin, E.J., and Boutte, C.C. (2020). The mycobacterial cell envelope—a moving target. Nat. Rev. Microbiol. *18*, 47–59.

Dupont, C., Viljoen, A., Dubar, F., Blaise, M., Bernut, A., Pawlik, A., Bouchier, C., Brosch, R., Guérardel, Y., Lelièvre, J., et al. (2016). A new piperidinol derivative targeting mycolic acid transport in *Mycobacterium abscessus*. Mol. Microbiol. *101*, 515–529.

Dupont, C., Chen, Y., Xu, Z., Roquet-Banères, F., Blaise, M., Witt, A.-K., Dubar, F., Biot, C., Guérardel, Y., Maurer, F.P., et al. (2019). A piperidinol-containing molecule is active against *Mycobacterium tuberculosis* by inhibiting the mycolic acid flippase activity of MmpL3. J. Biol. Chem. *294*, 17512–17523.

Emsley, P., Lohkamp, B., Scott, W.G., and Cowtan, K. (2010). Features and development of Coot. Acta Crystallogr. D: Biol. Crystallogr. *66*, 486–501.

Foss, M.H., Pou, S., Davidson, P.M., Dunaj, J.L., Winter, R.W., Pou, S., Licon, M.H., Doh, J.K., Li, Y., Kelly, J.X., et al. (2016). Diphenylether-modified 1,2-diamines with improved drug properties for development against *Mycobacterium tuberculosis*. ACS Infect. Dis. *2*, 500–508.

Fujita, Y., Naka, T., Doi, T., and Yano, I. (2005). Direct molecular mass determination of trehalose monomycolate from 11 species of mycobacteria by MALDI-TOF mass spectrometry. Microbiology *151*, 1443–1452.

Goldman, R.C. (2013). Why are membrane targets discovered by phenotypic screens and genome sequencing in *Mycobacterium tuberculosis*? Tuberculosis *93*, 569–588.

Goodsell, D.S., Autin, L., and Olson, A.J. (2019). Illustrate: software for biomolecular illustration. Structure *27*, 1716–1720.e1.

De Groote, M.A., Jackson, M., Gonzalez-Juarrero, M., Li, W., Young, C.L., Wong, C., Graham, J., Day, J., Hoang, T., Jarvis, T.C., et al. (2018). Optimization and lead selection of benzothiazole amide analogs toward a novel antimycobacterial agent. Front. Microbiol. *9*, 2231.

Grover, S., Engelhart, C.A., Pérez-Herrán, E., Li, W., Abrahams, K.A., Papavinasasundaram, K., Bean, J.M., Sassetti, C.M., Mendoza-Losana, A., Besra, G.S., et al. (2021). Two-way regulation of MmpL3 expression identifies and validates inhibitors of MmpL3 function in *Mycobacterium tuberculosis*. ACS Infect. Dis. *7*, 141–152.

Grzegorzewicz, A.E., Pham, H., Gundi, V.A.K.B., Scherman, M.S., North, E.J., Hess, T., Jones, V., Gruppo, V., Born, S.E.M., Kordulakova, J., et al. (2012). Inhibition of mycolic acid transport across the *Mycobacterium tuberculosis* plasma membrane. Nat. Chem. Biol. *8*, 334–341.

Guerra-Assunçaõ, J.A., Houben, R.M.G.J., Crampin, A.C., Mzembe, T., Mallard, K., Coll, F., Khan, P., Banda, L., Chiwaya, A., Pereira, R.P.A., et al. (2015). Recurrence due to relapse or reinfection with *Mycobacterium tuberculosis*: a whole-genome sequencing approach in a large, population-based cohort with a high HIV infection prevalence and active follow-up. J. Infect. Dis. *211*, 1154–1163.

Hunt, M., Bradley, P., Lapierre, S.G., Heys, S., Thomsit, M., Hall, M.B., Malone, K.M., Wintringer, P., Walker, T.M., Cirillo, D.M., et al. (2019). Antibiotic resistance prediction for *Mycobacterium tuberculosis* from genome sequence data with Mykrobe. Wellcome Open Res. *4*, 191.

Ioerger, T.R., O'Malley, T., Liao, R., Guinn, K.M., Hickey, M.J., Mohaideen, N., Murphy, K.C., Boshoff, H.I.M., Mizrahi, V., Rubin, E.J., et al. (2013). Identification of new drug targets and resistance mechanisms in *Mycobacterium tuberculosis*. PLoS One *8*, e75245.

Jackson, M. (2014). The mycobacterial cell envelope—lipids. CSH Perspect. Med. *4*, 1–22.

Jurcik, A., Bednar, D., Byska, J., Marques, S.M., Furmanova, K., Daniel, L., Kokkonen, P., Brezovsky, J., Strnad, O., Stourac, J., et al. (2018). CAVER Analyst 2.0: analysis and visualization of channels and tunnels in protein structures and molecular dynamics trajectories. Bioinformatics *34*, 3586–3588.

Kelley, L.A., Mezulis, S., Yates, C., Wass, M., and Sternberg, M. (2015). The Phyre2 web portal for protein modelling, prediction, and analysis. Nat. Protoc. *10*, 845–858.

Klenotic, P.A., Moseng, M.A., Morgan, C.E., and Yu, E.W. (2020). Structural and functional diversity of resistance-nodulation-cell division transporters. Chem. Rev. *121*, 5378–5416.

Korycka-Machała, M., Viljoen, A., Pawełczyk, J., Borówka, P., Dziadek, B., Gobis, K., Brzostek, A., Kawka, M., Blaise, M., Strapagiel, D., et al. (2019). 1H-Benzo[*d*]Imidazole derivatives affect MmpL3 in *Mycobacterium tuberculosis*. Antimicrob. Agents Chemother. *63*, e00441–19.

Kozikowski, A.P., Onajole, O.K., Stec, J., Dupont, C., Viljoen, A., Richard, M., Chaira, T., Lun, S., Bishai, W., Raj, V.S., et al. (2017). Targeting mycolic acid transport by indole-2-carboxamides for the treatment of *Mycobacterium abscessus* infections. J. Med. Chem. *60*, 5876–5888.

Li, W., Obregón-Henao, A., Wallach, J.B., North, E.J., Lee, R.E., Gonzalez-Juarrero, M., Schnappinger, D., and Jackson, M. (2016). Therapeutic potential of the *Mycobacterium tuberculosis* mycolic acid transporter, Mmpl3. Antimicrob. Agents Chemother. *60*, 5198–5207.

Li, W., Sanchez-Hidalgo, A., Jones, V., de Moura, V.C.N., North, E.J., and Jackson, M. (2017). Synergistic interactions of MmpL3 inhibitors with antitubercular compounds *in vitro*. Antimicrob. Agents Chemother. *61*, 1–6.

Li, W., Yazidi, A., Pandya, A.N., Hegde, P., Tong, W., Calado Nogueira de Moura, V., North, E.J., Sygusch, J., and Jackson, M. (2018). MmpL3 as a target for the treatment of drug-resistant nontuberculous mycobacterial infections. Front. Microbiol. *9*, 1547.

Li, W., Stevens, C.M., Pandya, A.N., Darzynkiewicz, Z., Bhattarai, P., Tong, W., Gonzales-Juarrero, M., North, E.J., Zgurskaya, H.I., and Jackson, M.C. (2019). Direct inhibition of MmpL3 by novel antitubercular compounds. ACS Infect. Dis. *5*, 1001–1012.

Li, M., Phua, Z.Y., Xi, Y., Xu, Z., Nyantakyi, S.A., Li, W., Jackson, M., Wong, M.W., Lam, Y., Chng, S.S., et al. (2020). Potency increase of spiroketal analogs of membrane inserting indolyl mannich base antimycobacterials is due to acquisition of MmpL3 inhibition. ACS Infect. Dis. *6*, 1882–1893.

Liebschner, D., Afonine, P.v., Baker, M.L., Bunkoczi, G., Chen, V.B., Croll, T.I., Hintze, B., Hung, L.W., Jain, S., McCoy, A.J., et al. (2019). Macromolecular structure determination using X-rays, neutrons and electrons: recent developments in Phenix. Acta Crystallogr. D Struct. Biol. *75*, 861–877.

Lun, S., Guo, H., Onajole, O.K., Pieroni, M., Gunosewoyo, H., Chen, G., Tipparaju, S.K., Ammerman, N.C., Kozikowski, A.P., and Bishai, W.R. (2013). Indoleamides are active against drug-resistant *Mycobacterium tuberculosis*. Nat. Commun. *4*, 1–8.

Madeira, F., Park, Y.M., Lee, J., Buso, N., Gur, T., Madhusoodanan, N., Basutkar, P., Tivey, A.R.N., Potter, S.C., Finn, R.D., et al. (2019). The EMBL-EBI search and sequence analysis tools APIs in 2019. Nucleic Acids Res. *47*, W636–W641.

McNeil, M.B., Dennison, D., and Parish, T. (2017). Mutations in MmpL3 alter membrane potential, hydrophobicity and antibiotic susceptibility in *Mycobacterium smegmatis*. Microbiology *163*, 1065–1070.

McNeil, M.B., Malley, T.O., Dennison, D., Shelton, C.D., and Sunde, B. (2020). Multiple mutations in *Mycobacterium tuberculosis* MmpL3 Increase resistance to MmpL3 inhibitors. mSphere *5*, 1–7.

Molin, M.D., Selchow, P., Schäfle, D., Tschumi, A., Ryckmans, T., Laage-witt, S., and Sander, P. (2019). Identification of novel scaffolds targeting *Mycobacterium tuberculosis*. J. Mol. Med. *97*, 1601–1613.

Nikaido, H. (2018). RND transporters in the living world. Res. Microbiol. *169*, 363–371.

Nikonenko, B.V., Protopopova, M., Samala, R., Einck, L., and Nacy, C.A. (2007). Drug therapy of experimental tuberculosis (TB): improved outcome by combining SQ109, a new diamine antibiotic, with existing TB drugs. Antimicrob. Agents Chemother. *51*, 1563–1565.

Pandya, A.N., Prathipati, P.K., Hegde, P., Li, W., Graham, K.F., Mandal, S., Drescher, K.M., Destache, C.J., Ordway, D., Jackson, M., et al. (2019). Indole-2-carboxamides are active against *Mycobacterium abscessus* in a mouse model of acute infection. Antimicrob. Agents Chemother. *63*, 1–16.

Pankhurst, L.J., del Ojo Elias, C., Votintseva, A.A., Walker, T.M., Cole, K., Davies, J., Fermont, J.M., Gascoyne-Binzi, D.M., Kohl, T.A., Kong, C., et al. (2016). Rapid, comprehensive, and affordable mycobacterial diagnosis with whole-genome sequencing: a prospective study. Lancet Respir. Med. *4*, 49–58.

Pettersen, E.F., Goddard, T.D., Huang, C.C., Couch, G.S., Greenblatt, D.M., Meng, E.C., and Ferrin, T.E. (2004). UCSF Chimera—a visualization system for exploratory research and analysis. J. Comput. Chem. *25*, 1605–1612.

Pettersen, E.F., Goddard, T.D., Huang, C.C., Meng, E.C., Couch, G.S., Croll, T.I., Morris, J.H., and Ferrin, T.E. (2021). UCSF ChimeraX: structure visualization for researchers, educators, and developers. Protein Sci. *30*, 70–82.

Poce, G., Bates, R.H., Alfonso, S., Cocozza, M., Porretta, G.C., Ballell, L., Rullas, J., Ortega, F., de Logu, A., Agus, E., et al. (2013). Improved BM212 MmpL3 inhibitor analogue shows efficacy in acute murine model of tuberculosis infection. PLoS One *8*, e56980.

Poce, G., Consalvi, S., Venditti, G., Alfonso, S., Desideri, N., Fernandez-Menendez, R., Bates, R.H., Ballell, L., Barros Aguirre, D., Rullas, J., et al. (2019). Novel pyrazole-containing compounds active against *Mycobacterium tuberculosis*. ACS Med. Chem. Lett. *10*, 1423–1429.

Punjani, A., Rubinstein, J.L., Fleet, D.J., and Brubaker, M.A. (2017). cryoSPARC: algorithms for rapid unsupervised cryo-EM structure determination. Nat. Methods *14*, 290–296.

Punjani, A., Zhang, H., and Fleet, D.J. (2020). Non-uniform refinement: adaptive regularization improves single-particle cryo-EM reconstruction. Nat. Methods *17*, 1214–1221.

Rao, S.P.S., Lakshminarayana, S.B., Kondreddi, R.R., Herve, M., Camacho, L.R., Bifani, P., Kalapala, S.K., Jiricek, J., Ma, N.L., Tan, B.H., et al. (2013). Indolcarboxamide is a preclinical candidate for treating multidrug-resistant tuberculosis. Sci. Transl. Med. *5*, 214ra168.

Rayasam, G.V. (2014). MmpL3 a potential new target for development of novel anti-tuberculosis drugs. Expert Opin. Ther. Targets *18*, 247–256.

Raynaud, C., Daher, W., Johansen, M.D., Roquet-Banères, F., Blaise, M., Onajole, O.K., Kozikowski, A.P., Herrmann, J.L., Dziadek, J., Gobis, K., et al. (2020). Active benzimidazole derivatives targeting the MmpL3 transporter in *Mycobacterium abscessus*. ACS Infect. Dis. *6*, 324–337.

Remuiñán, M.J., Pérez-Herrán, E., Rullás, J., Alemparte, C., Martínez-Hoyos, M., Dow, D.J., Afari, J., Mehta, N., Esquivias, J., Jiménez, E., et al. (2013). Tetrahydropyrazolo[1,5-a]pyrimidine-3-carboxamide and N-benzyl-6',7'-dihydrospiro[piperidine-4,4'-thieno[3,2-c]pyran] analogues with bactericidal efficacy against *Mycobacterium tuberculosis* targeting MmpL3. PLoS One *8*, e60933.

La Rosa, V., Poce, G., Canseco, J.O., Buroni, S., Pasca, M.R., Biava, M., Raju, R.M., Porretta, G.C., Alfonso, S., Battilocchio, C., et al. (2012). MmpL3 is the cellular target of the antitubercular pyrrole derivative BM212. Antimicrob. Agents Chemother. *56*, 324–331.

Shao, M., McNeil, M., Cook, G.M., and Lu, X. (2020). MmpL3 inhibitors as antituberculosis drugs. Eur. J. Med. Chem. *200*, 112390.

Shetty, A., Xu, Z., Lakshmanan, U., Hill, J., Choong, M.L., Chng, S.-S., Yamada, Y., Poulsen, A., Dick, T., and Gengenbacher, M. (2018). Novel acetamide indirectly targets mycobacterial transporter MmpL3 by proton motive force disruption. Front. Microbiol. *9*, 2960.

Stanley, S.A., Grant, S.S., Kawate, T., Iwase, N., Shimizu, M., Wivagg, C., Silvis, M., Kazyanskaya, E., Aquadro, J., Golas, A., et al. (2012). Identification of novel inhibitors of *M. tuberculosis* growth using whole cell based high-throughput screening. ACS Chem. Biol. *7*, 1377–1384.

Stec, J., Onajole, O.K., Lun, S., Guo, H., Merenbloom, B., Vistoli, G., Bishai, W.R., and Kozikowski, A.P. (2016). Indole-2-carboxamide-based MmpL3 inhibitors show exceptional antitubercular activity in an animal model of tuberculosis infection. J. Med. Chem. *59*, 6232–6247.

Su, C.C., Klenotic, P.A., Bolla, J.R., Purdy, G.E., Robinson, C.V., and Yu, E.W. (2019). MmpL3 is a lipid transporter that binds trehalose monomycolate and phosphatidylethanolamine. Proc. Natl. Acad. Sci. U S A *166*, 11241–11246.

Székely, R., and Cole, S.T. (2016). Mechanistic insight into mycobacterial MmpL protein function. Mol. Microbiol. *99*, 831–834.

CellPress
OPEN ACCESS

Tahlan, K., Wilson, R., Kastrinsky, D.B., Arora, K., Nair, V., Fischer, E., Whitney Barnes, S., Walker, J.R., Alland, D., Barry, C.E., et al. (2012). SQ109 targets MmpL3, a membrane transporter of trehalose monomycolate involved in mycolic acid donation to the cell wall core of *Mycobacterium tuberculosis*. Antimicrob. Agents Chemother. *56*, 1797–1809.

Tantry, S.J., Degiacomi, G., Sharma, S., Jena, L.K., Narayan, A., Guptha, S., Shanbhag, G., Menasinakai, S., Mallya, M., Awasthy, D., et al. (2015). Whole cell screen based identification of spiropiperidines with potent antitubercular properties. Bioorg. Med. Chem. Lett. *25*, 3234–3245.

The CRyPTIC Consortium (2021). Epidemiological cutoff values.for a 96-well broth microdilution plate for high-throughput research antibiotic susceptibility testing of *M. tuberculosis*. medRxiv. https://doi.org/10.1101/2021.02.24.21252386.

The CRyPTIC Consortium and the 100,000 genomes project (2018). Prediction of susceptibility to first-line tuberculosis drugs by DNA sequencing. New Engl. J. Med. *379*, 1403–1415.

Trofimov, V., Kicka, S., Mucaria, S., Hanna, N., Ramon-Olayo, F., del Peral, L.V.-G., Lelièvre, J., Ballell, L., Scapozza, L., Besra, G.S., et al. (2018). Antimycobacterial drug discovery using mycobacteria-infected amoebae identifies anti-infectives and new molecular targets. Sci. Rep. *8*, 3939.

Udwadia, Z.F., Amale, R.A., Ajbani, K.K., and Rodrigues, C. (2012). Totally drug-resistant tuberculosis in India. Clin. Infect. Dis. *54*, 579–581.

Varela, C., Rittmann, D., Singh, A., Krumbach, K., Bhatt, K., Eggeling, L., Besra, G.S., and Bhatt, A. (2012). MmpL genes are associated with mycolic acid metabolism in mycobacteria and corynebacteria. Chem. Biol. *19*, 498–506.

Velayati, A.A., Masjedi, M.R., Farnia, P., Tabarsi, P., Ghanavi, J., ZiaZarifi, A.H., and Hoffner, S.E. (2009). Emergence of new forms of totally drug-resistant tuberculosis bacilli: super extensively drug-resistant tuberculosis or totally drug-resistant strains in Iran. Chest *136*, 420–425.

Viljoen, A., Dubois, V., Girard-Misguich, F., Blaise, M., Herrmann, J.L., and Kremer, L. (2017). The diverse family of MmpL transporters in mycobacteria: from regulation to antimicrobial developments. Mol. Microbiol. *104*, 889–904.

Waldo, G.S., Standish, B.M., Berendzen, J., and Terwilliger, T.C. (1999). Rapid protein-folding assay using green fluorescent protein. Nat. Biotechnol. *17*, 691–695.

Walker, T.M., Kohl, T.A., Omar, S.V., Hedge, J., del Ojo Elias, C., Bradley, P., Iqbal, Z., Feuerriegel, S., Niehaus, K.E., Wilson, D.J., et al. (2015). Whole-genome sequencing for prediction of *Mycobacterium tuberculosis* drug susceptibility and resistance: a retrospective cohort study. Lancet Infect. Dis. *15*, 1193–1202.

Williams, C.J., Headd, J.J., Moriarty, N.W., Prisant, M.G., Videau, L.L., Deis, L.N., Verma, V., Keedy, D.A., Hintze, B.J., Chen, V.B., et al. (2018). MolProbity: more and better reference data for improved all-atom structure validation. Protein Sci. *27*, 293–315.

Williams, J.T., Haiderer, E.R., Coulson, G.B., Conner, K.N., Ellsworth, E., Chen, C., Alvarez-Cabrera, N., Li, W., Jackson, M., Dick, T., et al. (2019). Identification of new MmpL3 inhibitors by untargeted and targeted mutant screens defines MmpL3 domains with differential resistance. Antimicrob. Agents Chemother. *63*, 1–18.

World Health Organization (2020). Global Tuberculosis Report 2020. Licence: CC BY-NC-SA 3.0. LGO (Geneva: World Health Organization).

Yang, X., Hu, T., Yang, X., Xu, W., Yang, H., Guddat, L.W., Zhang, B., and Rao, Z. (2020). Structural basis for the inhibition of mycobacterial MmpL3 by NITD-349 and SPIRO. J. Mol. Biol. *432*, 4426–4434.

Zhang, B., Li, J., Yang, X., Wu, L., Zhang, J., Yang, Y., Zhao, Y., Zhang, L., Yang, X., Yang, X., et al. (2019). Crystal structures of membrane transporter MmpL3, an anti-TB drug target. Cell *176*, 636–648.e13.

Zheng, H., Williams, J.T., Coulson, G.B., Haiderer, E.R., and Abramovitch, R.B. (2018). HC2091 kills *Mycobacterium tuberculosis* by targeting the MmpL3 mycolic acid transporter. Antimicrob. Agents Chemother. *62*, e02459–17.

Zivanov, J., Nakane, T., and Scheres, S.H.W. (2019). A Bayesian approach to beam-induced motion correction in cryo-EM single-particle analysis. IUCrJ *6*, 5–17.

Zivanov, J., Nakane, T., and Scheres, S.H.W. (2020). Estimation of high-order aberrations and anisotropic magnification from cryo-EM data sets in RELION-3.1. IUCrJ *7*, 253–267.

STAR★METHODS

KEY RESOURCES TABLE

REAGENT or RESOURCE	SOURCE	IDENTIFIER
**Bacterial and virus strains**		
E. coli OmniMAX	Invitrogen	Cat# C854003
E. coli C43(DE3)	Lucigen	Cat# 60446-1
**Chemicals, peptides, and recombinant proteins**		
Lauryl Maltose Neopentyl Glycol (LMNG)	Anatrace	Cat# NG310
TEV Protease	Produced In-house	N/A
**Critical commercial assays**		
HisPur-Ni-NTA Resin	Thermo Fisher Scientific	Cat# 88221
HisTrap Column	Cytiva	Cat# 17-5255-01
Superdex 200 10/300 Increase GL Column	Cytiva	Cat# 28-9909-44
**Deposited data**		
Mtb MmpL3$_{1-753}$ Cryo-EM Volume	This Paper	EMDB code: EMD-12604
Mtb MmpL3$_{1-753}$ Atomic Coordinates	This Paper	PDB code: 7NVH
Msmg MmpL3$_{1-773}$ Atomic Coordinates	Su et al., 2019	PDB code: 6OR2
**Oligonucleotides**		
Primer: NdeI MmpL3$_{1-753}$ Forward GGAATTCCATATGTTTGCATGGT GGGGTCG	This Paper	N/A
Primer: BamHI MmpL3$_{1-753}$ Reverse ACTGGGATCCATCAGGCAGATGA ATTTCACCCAGGCC	This Paper	N/A
**Recombinant DNA**		
Plasmid: pMA-T-MmpL3$_{1-944}$	Invitrogen GeneArt	N/A
Plasmid: pWaldo-GFPd	Drew et al., 2001	N/A
Plasmid: pWaldo-GFPd-MmpL3$_{1-753}$	This Paper	N/A
**Software and algorithms**		
SIMPLE 3.0	Caesar et al., 2020	https://simplecryoem.com/
cryoSPARC (v.2.15.0)	Punjani et al., 2017	https://cryosparc.com/
UCSF pyem (v.0.5)	Asarnow et al., 2019	https://zenodo.org/record/3576630
RELION 3.1.0	Zivanov et al., 2020	https://www3.mrc-lmb.cam.ac.uk/relion/index.php/Main_Page
Phyre2 Server	Kelley et al., 2015	http://www.sbg.bio.ic.ac.uk/phyre2/html/page.cgi?id=index
Coot (v.0.9.2-pre)	Emsley et al., 2010	https://www2.mrc-lmb.cam.ac.uk/personal/pemsley/coot/
PHENIX (v.1.18.2)	Liebschner et al., 2019	http://www.phenix-online.org/
UCSF Chimera (v.1.13.1)	Pettersen et al., 2004	https://www.cgl.ucsf.edu/chimera/
UCSF ChimeraX (v.1.1)	Pettersen et al., 2021	https://www.rbvi.ucsf.edu/chimerax/
CAVER Analyst 2.0	Jurcik et al., 2018	http://www.caver.cz/
ConSurf Server	Ashkenazy et al., 2016	https://consurf.tau.ac.il/
T-Coffee	Madeira et al., 2019	https://www.ebi.ac.uk/Tools/msa/tcoffee/
ChemDraw Professional (v.17.0.0.206)	PerkinElmer Informatics	https://perkinelmerinformatics.com/products/research/chemdraw/
Illustrate	Goodsell et al., 2019	https://ccsb.scripps.edu/illustrate/
TEXshade	Beitz, 2000a	https://ctan.org/pkg/texshade

(*Continued on next page*)

Continued

REAGENT or RESOURCE	SOURCE	IDENTIFIER
T_EXtopo	Beitz, 2000b	https://ctan.org/pkg/textopo
Mykrobe	Hunt et al., 2019	https://www.mykrobe.com/
Other		
Vivaspin-20 (50 kDa MWCO) Concentrator	Sartorius	Cat# VS2032
Vivaspin-500 (50 kDa MWCO) Concentrator	Sartorius	Cat# VS0132
Quantifoil 300 Mesh Au R1.2/1.3 Grids	Agar Scientific	Cat# AGS143-8

RESOURCE AVAILABILITY

Lead contact

Further information and requests for resources and reagents should be directed to and will be fulfilled by the lead contact, Simon Newstead (simon.newstead@bioch.ox.ac.uk).

Materials availability

All unique reagents generated in this study are available from the lead contact upon reasonable request.

Data and code availability

The cryo-EM volume has been deposited in the Electron Microscopy DataBank (EMDB) with accession code EMDB-12604, and the atomic coordinates have been deposited in the Protein DataBank (PDB) with accession code 7NVH. The mutational data underlying Figures 3 and 4 are provided in Tables S2 and S3 respectively. This paper does not report original code. Any additional information required to reanalyze the data reported is available from the lead contact upon request.

EXPERIMENTAL MODEL AND SUBJECT DETAILS

Plasmid propagation and clone recovery were performed using OmniMAX *E. coli* cells (Invitrogen) plated on Luria Broth (LB) agar (37°C) or inoculated in LB liquid media (37°C, 180 RPM); both grown overnight in the presence of a selective antibiotic. Recombinant *Mtb* MmpL3_{1-753} was produced in *E. coli* C43(DE3) cells (Lucigen), cultured in Terrific Broth supplemented with kanamycin (50 μg mL^{-1}) for ~18 hr (25°C, 215 RPM) following IPTG induction (final concentration 0.4 mM) at an OD_{600} ~0.6–0.8.

METHOD DETAILS

Mtb MmpL3_{1-753} molecular cloning & purification

An *E. coli* codon-optimised version of the gene (*Rv0206c*) encoding full-length (residues 1–944) *Mtb* MmpL3 (Uniprot ID: P9WJV5) was synthesised by Invitrogen GeneArt services, provided in the vector pMA-T. Using the primers detailed in the Key Resources Table, a truncated open reading frame encoding only residues 1–753 of *Mtb* MmpL3 was amplified and then restriction sub-cloned into IPTG-inducible vector pWaldo-GFPd (Drew et al., 2001; Waldo et al., 1999); placing it in-frame with a C-terminal TEV-GFP-His_8 affinity-tag. MmpL3_{1-753} expression in *E. coli* and purification to homogeneity, by Ni^{2+}-affinity chromatography followed by SEC, was achieved using standard protocols (Drew et al., 2005). All purification steps were undertaken at 4°C. Briefly, thawed membranes were first resuspended in 1X PBS supplemented with 150 mM NaCl, prior to solubilization in 1% (w/v) LMNG (Anatrace) under gentle agitation for 1.5 hr. Non-solubilized material was removed by ultracentrifugation (>200,000 g, 1 hr), and then imidazole to a final concentration of 15 mM was added to the recovered supernatant. Protein in this was bound to HisPur-Ni-NTA resin (Thermo Fisher Scientific) in batch, by incubation for ~2 hr 45 min with stirring. The resin, packed into a glass econo-column (BioRad), was next sequentially washed with 10 column volumes (CVs) of purification buffer (1x PBS, 150 mM NaCl, 0.1% (w/v) LMNG) containing 15 mM imidazole, and 15 CVs supplemented with 30 mM imidazole. MmpL3_{1-753} was finally eluted in 5 CVs of purification buffer featuring 250 mM imidazole. After TEV protease addition, the protein was dialyzed overnight in gel filtration buffer (20 mM Tris pH 7.5, 150 mM NaCl, 0.003% (w/v) LMNG). The next day, dialysate was passed through a 5 mL HisTrap column (Cytiva) to remove both the TEV protease and liberated GFP-His_8 affinity-tag. The resulting pure MmpL$_{1-753}$ was spin concentrated using a Vivaspin-20 (50 kDa MWCO, Sartorius), prior to SEC polishing on a Superdex 200 10/300 Increase GL column (Cytiva) pre-equilibrated in gel filtration buffer. For cryo-EM, SEC fractions of interest were immediately pooled, spin concentrated to 2 mg mL^{-1} in a Vivaspin-500 (50 kDa MWCO, Sartorius), and the protein then kept on ice until grid preparation the same day. MmpL3_{1-753} purity was assessed throughout purification by analysis of samples loaded on 10% SDS-PAGE gels stained with Coomassie blue (InstantBlue, Expedeon).

Cryo-EM sample preparation & imaging

A 4 μL aliquot of pure, LMNG-solubilized, MmpL3_{1-753} (2 mg mL^{-1}) was dispensed onto the surface of a freshly glow-discharged holey carbon-coated grid (Quantifoil 300 mesh, Au R1.2/1.3, Agar Scientific). After allowing 10 s for adsorption, the grid was blotted

for 3 s (blot force −5, 100% humidity, 8°C) and then flash-frozen by plunging into liquid ethane using a Vitrobot Mark IV (Thermo Fisher Scientific). Data were gathered in counting super-resolution mode on a 300 kV Titan Krios G3 cryo-TEM (Thermo Fisher Scientific) equipped with a BioQuantum Imaging Filter (Gatan) and K3 Direct Electron Detector (Gatan). Using a pixel size 0.832 Å, 15,020 movies spanning a defocus range of −3.0 to −0.5 μm were collected; each with a total dose of 58.2 e−/Å^2, spread across 40 fractions, over an exposure time of 2.80 s. A representative micrograph is shown in Figure S1B.

Cryo-EM data processing

A schematic summarising the MmpL3$_{1-753}$ cryo-EM data processing workflow is provided in Figure S1C. Patched (15 x 10) motion correction, dose weighting, contrast transfer function (CTF) estimation, and particle autopicking were all implemented on-the-fly using SIMPLE 3.0 (Caesar et al., 2020). This yielded 7,764,838 particles, extracted in 256 × 256-pixel boxes, which were then subject to two rounds of reference-free 2D classification; the first on-the-fly within SIMPLE 3.0, using cluster2D-stream, and the second in cryoSPARC (v.2.15.0, Punjani et al., 2017). Discarding poor-quality classes after each round left 1,564,980 particles for downstream processing, which was performed predominantly in cryoSPARC. The particle subset was first subject to multi-class (k = 4) *ab initio* model generation, without symmetry imposition (i.e. in C1). This generated a single featureful volume, displaying the expected MmpL3$_{1-753}$ domain organization, composed of 758,680 particles. Subsequently passing these through two rounds of non-uniform refinement (Punjani et al., 2020), interspersed by Bayesian particle polishing (Zivanov et al., 2019) undertaken in RELION 3.1.0 (Zivanov et al., 2020) within 320 × 320-pixel boxes, gave a 3.4 Å map. The requisite STAR file for this polishing was generated using the csparc2star.py script from UCSF pyem (Asarnow et al., 2019). Further heterogeneous refinement of the volume against the previous four *ab initio* classes recovered 562,667 particles, whose non-uniform refinement returned a 3.3 Å reconstruction. A final round of reference-free 2D classification was employed to remove any remaining suboptimal particles, resulting in a stack retaining the best 414,082. Successive non-uniform, beamtilt, and local refinement of these yielded the 3.0 Å map of MmpL3$_{1-753}$ deposited in the EMDB under accession code EMD-12604. Throughout processing all refinement reference maps were lowpass filtered to 8 Å. Gold-standard Fourier shell correlations (FSC) using the 0.143 criterion, as well as local resolution estimations, were calculated in RELION 3.1.0 (Figures S1D and S1E).

Model building & refinement

Atomic model building began by threading the *Mtb* MmpL3$_{1-753}$ sequence onto a previously reported crystal structure of the *Msmg* orthologue (PDB code: 6OR2, Su et al., 2019), using the Phyre2 web server (Kelley et al., 2015). The threaded structure was then docked into the 3.0 Å cryo-EM volume in UCSF Chimera (v.1.13.1, Pettersen et al., 2004), prior to further rigid body fitting in Coot (v.0.9.2-pre, Emsley et al., 2010). Within the latter program the model underwent manual real-space refinement (RSR), regions lacking unambiguous density were removed (residues 343–377, 753), and an LMNG molecule was built into the splayed species occupying the PD chamber. To generate the final model, detailed in Table S1 and deposited in the PDB under accession code 7NVH, the MmpL3$_{1-753}$-detergent complex underwent further rounds of RSR in PHENIX (v.1.18.2, Liebschner et al., 2019) with secondary structure, rotamer, ligand, and Ramachandran restraints applied, alongside additional minor adjustments in Coot. The globally sharpened map, output from cryoSPARC local refinement, was used for all rounds of RSR in both Coot and PHENIX. Model validity was assessed by MolProbity (Williams et al., 2018) executed within the latter suite. The density fit for modeled TM side chains and the LMNG ligand are shown in Figure S1F.

Structural analysis & data Visualisation

The MmpL3$_{1-753}$ cavity was calculated with Caver Analyst 2.0 (Jurcik et al., 2018) using default parameters. Conservation analysis was performed on the ConSurf server (Ashkenazy et al., 2016). The MmpL3 ortholog sequence alignment was generated by T-Coffee (Madeira et al., 2019), and typeset with T$_E$Xshade (Beitz, 2000a). All structural figures were composed in UCSF Chimera (v.1.13.1, Pettersen et al., 2004) or UCSF ChimeraX (v.1.1, Pettersen et al., 2021). Primary sequence representations of the structurally unresolved regions of *Mtb* MmpL3 were made with T$_E$Xtopo (Beitz, 2000b). Antitubercular skeletal formulas were prepared in ChemDraw Professional (v.17.0.0.206), and the protein elements of the graphical abstract were produced using Illustrate (Goodsell et al., 2019).

Mtb MmpL3 mutation collation

Variants of *Mtb* MmpL3 observed to confer resistance to one or more preclinical agents were extracted from 29 publications. These were then manually curated to give Table S2, which includes references to all the original studies. Mutations identified in mycobacterial species other than *Mtb* were back-mapped by means of sequence alignments between the relevant MmpL3 orthologs. The broader mutational landscape of *Mtb* MmpL3 was sampled by retrieving all *Mtb* genomes deposited in the ENA at the time of study, together with those reported in Walker et al. (2015) and The CRyPTIC Consortium and the 100,000 Genomes Project, 2018. Totalling 45,878, the majority of these genomes underwent *Mtb* lineage classification using Mykrobe (Hunt et al., 2019) and the short reads were mapped onto version 3 of the H37Rv *Mtb* reference genome (NC_000962.3) and variants called using Clockwork v.0.8.3 as described elsewhere (The CRyPTIC Consortium, 2021). The CRyPTIC data warehouse was then interrogated for all non-synonymous substitutions in the MmpL3 open reading frame. The resultant 7,475 mutations, and any lineage associations of the most abundant, are compiled in Table S3.

QUANTIFICATION AND STATISTICAL ANALYSIS

Cryo-EM reconstruction and model building of *Mtb* MmpL3$_{1\text{-}753}$ was performed as described in the relevant 'Method details' sections, employing the software packages SIMPLE 3.0, cryoSPARC (v.2.15.0), RELION 3.1.0, Coot (v.0.9.2-pre), and PHENIX (v.1.18.2) as noted in the Key Resources Table. Final model statistics are given in Table S1.

Structure, Volume *29*

Supplemental Information

Cryo-EM structure and resistance landscape of *M. tuberculosis* MmpL3: An emergent therapeutic target

Oliver Adams, Justin C. Deme, Joanne L. Parker, the CRyPTIC Consortium, Philip W. Fowler, Susan M. Lea, and Simon Newstead

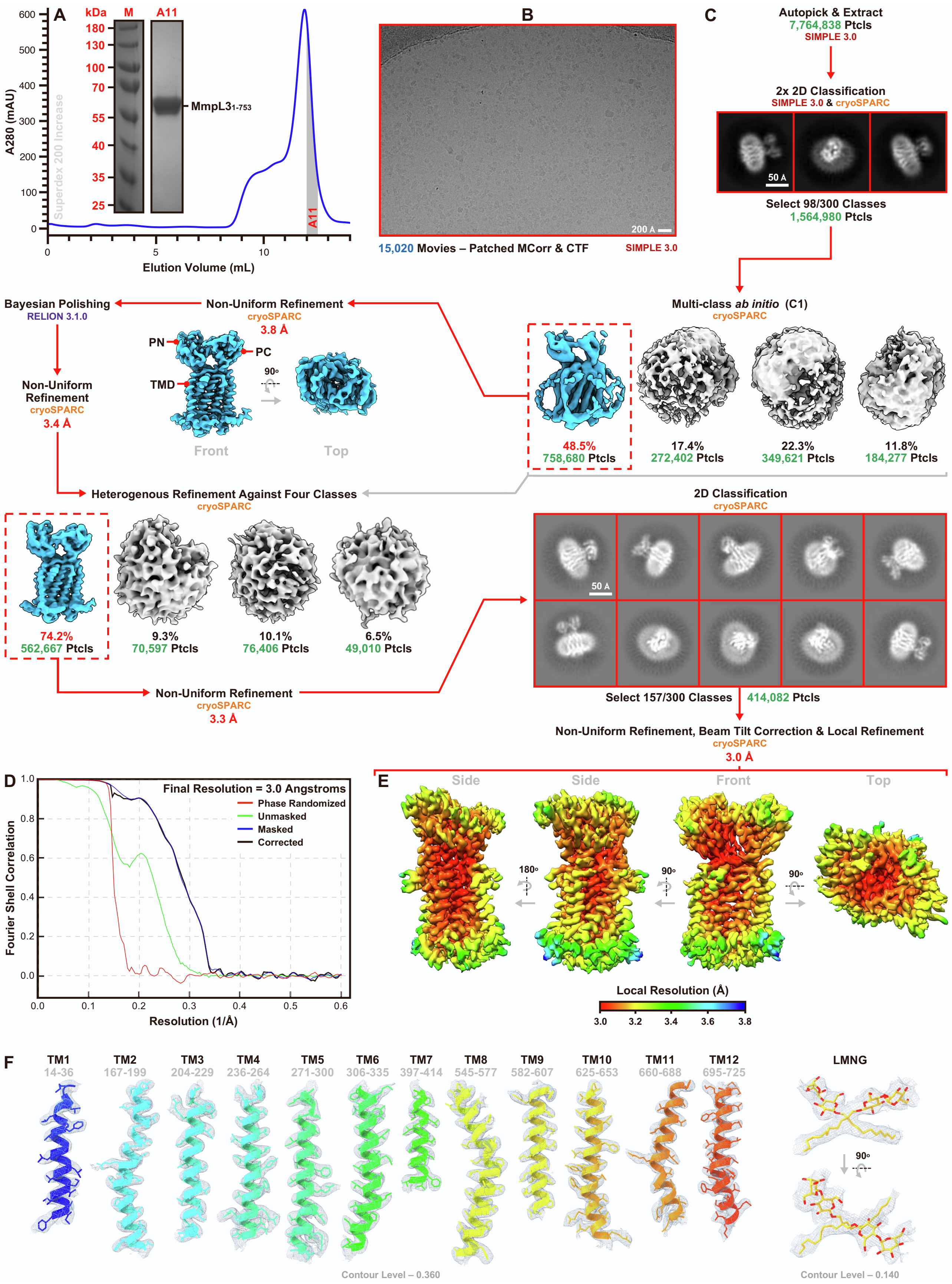

Figure S1. ***Mtb* MmpL3$_{1\text{-}753}$ Purification, Cryo-EM Workflow, Map Resolution and Model Density Fit, Related to Figure 1 and Table S1**

(A) Superdex 200 10/300 Increase GL profile recovered on size-exclusion chromatography (SEC) polishing of LMNG purified *Mtb* MmpL3$_{1\text{-}753}$ for cryo-EM studies. The grey bar denotes the SEC fraction ("A11") concentrated for imaging. The same material is analyzed on the inset Coomassie-stained SDS-PAGE gel lane, which confirms purification to homogeneity. Absorption at 280 nm (A280) is in milli-absorbance units (mAU). **(B)** Representative micrograph of the *Mtb* MmpL3$_{1\text{-}753}$ sample (2 mg mL^{-1}) collected for cryo-EM structure determination. Scale bar is 200 Å. **(C)** *Mtb* MmpL3$_{1\text{-}753}$ cryo-EM data processing workflow. The software package(s) employed at each stage are indicated. Scale bars are 50 Å. **(D)** Fourier shell correlation (FSC) plot for the final *Mtb* MmpL3$_{1\text{-}753}$ reconstruction. The stated global resolution is reported at the gold-standard 0.143 cut-off. **(E)** Local resolution estimates for the sharpened *Mtb* MmpL3$_{1\text{-}753}$ map, viewed from four directions. Micellar density is hidden. **(F)** Density fit for the 12 modelled *Mtb* MmpL3$_{1\text{-}753}$ transmembrane helices (TMs) as well as the LMNG molecule occupying the periplasmic vestibule, both colored as in Figure 1B.

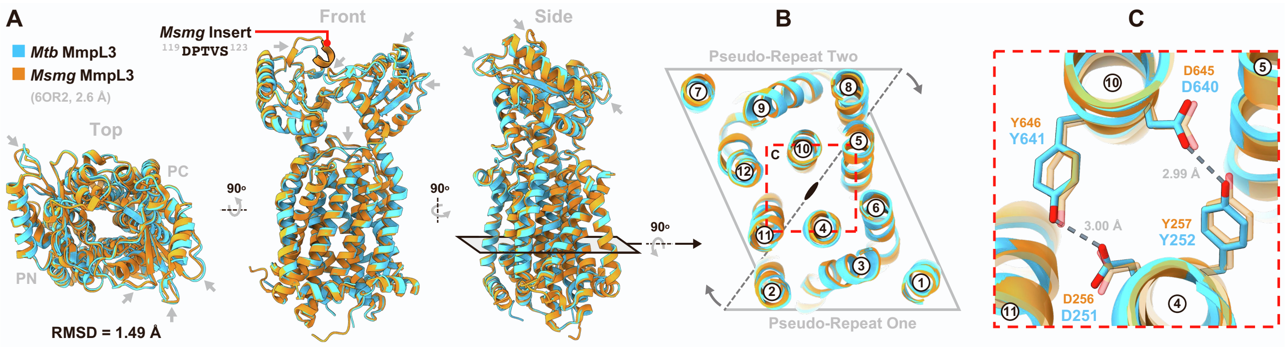

Figure S2. Structural Comparisons of *Mtb* MmpL3$_{1\text{-}753}$ and *Msmg* MmpL3, Related to Figure 1

(A) Alignment of the *Mtb* MmpL3$_{1\text{-}753}$ (blue) and *Msmg* MmpL3$_{1\text{-}773}$ (orange, PDB code: 6OR2, Su et al., 2019) models (1.49 Å RMSD over all atoms), shown in cartoon representation, and viewed from the membrane plane (front, side) or from the periplasm (top). Grey arrows emphasise deviations in loop architecture between the orthologs. A black outline, and accompanying annotation, identifies the PN pentapeptide (DPTVS) present in *Msmg* but not *Mtb* MmpL3. **(B)** Clipped view of the overlayed *Mtb* and *Msmg* MmpL3 transmembrane domains (TMDs), sliced as indicated in (A). The black oval designates the central 2-fold pseudo-symmetry axis, relating the two six transmembrane helix (TM) repeats (enclosed in grey triangles) that comprise the TMD. TMs are numbered. **(C)** Detailed close-up of region boxed in (B), showing the pair of Asp-Tyr dyads connecting TM4 and TM10 through hydrogen bonds (dashed lines) in the middle of the TMD. Colored as in (A), with side chains in stick form. Almost universally conserved in MmpLs, these residues are thought to be crucial for proton-motive force (PMF) transduction within the family. Notably, preclinical antituberculars targeting MmpL3 disrupt this Asp-Tyr hydrogen bond network; presumably de-energizing the transporter.

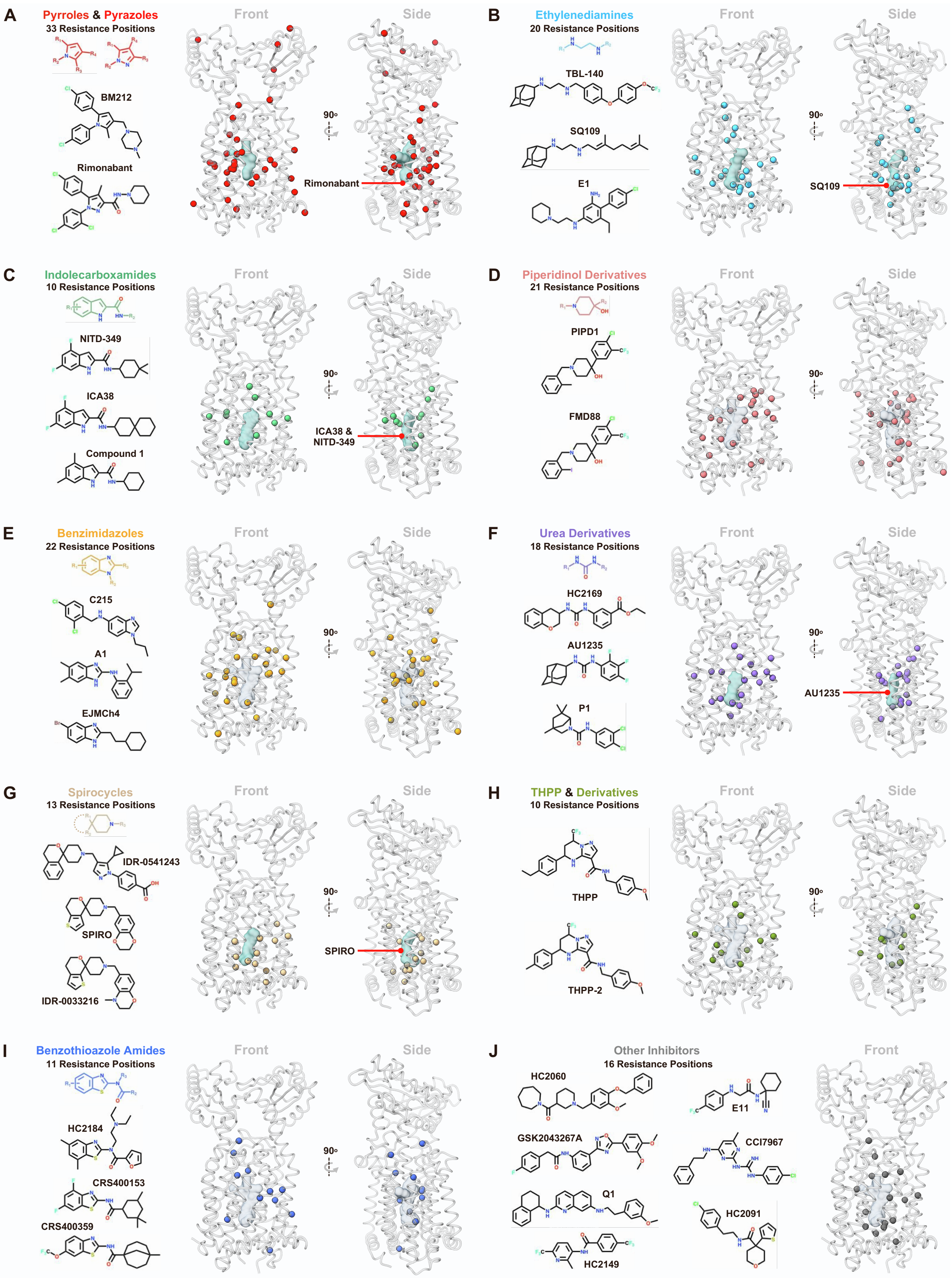

Figure S3. **Structural Mapping of MmpL3 Drug-Resistance Mutations Stratified by Inhibitor Class, Related to Figure 3 and Table S2**

(A – J) Discrete mapping, onto the *Mtb* $MmpL3_{1\text{-}753}$ structure, of collections of variants reported to confer resistance to members of each of the nine established classes of preclinical MmpL3 inhibitor (A – I), alongside a tenth miscellaneous grouping of predominantly orphan scaffolds (J). In each case resistance positions (RPs) are depicted by balls and the protein (transparent grey, licorice representation) viewed from one or more directions in the plane of the membrane. Where molecules of a given class have been co-crystallized with the *Msmg* homolog (PDB codes: 6AJG to 6AJJ, 7C2N & 7C2M, Zhang et al., 2019; Yang et al., 2020) a surface representation of the bound compound or compounds is superimposed (transparent teal) and annotated, otherwise the composite drug pocket is shown for reference (transparent grey, as in Figure 1C). Examples of agents affected by the plotted RPs are displayed for each family, and where informative a skeletal formula of the core class scaffold provided (R-groups denote variable substituents). Panels show RPs against (A) pyrrole/pyrazole, (B) ethylenediamine, (C) indolecarboxamide, (D) piperidinol derivative, (E) benzimidazole, (F) urea derivative, (G) spirocycle, (H) THPP associated, (I) benzothioazole amide, and (J) miscellaneous class MmpL3 inhibitors. All mutations are collated in Table S2.

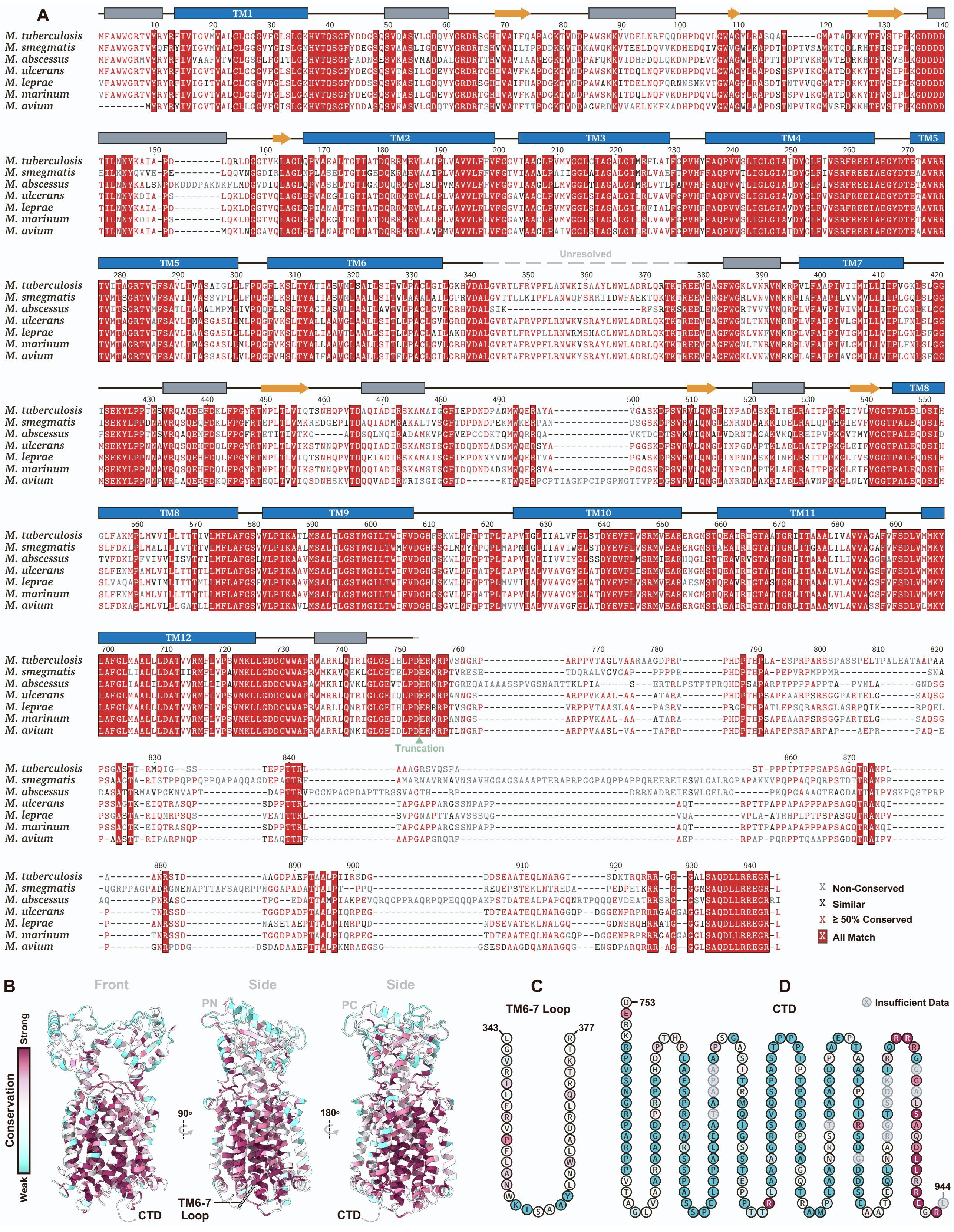

Figure S4. **Ortholog Sequence Alignment and Conservation Analysis, Related to Figures 3 and 4**

(A) Sequence alignment of *Mtb* MmpL3 with orthologs from *Msmg*, *M. abscessus*, *M. ulcerans*, *M. leprae*, *M. marinum*, and *M. avium*. Positions of secondary structure elements, derived from the *Mtb* $MmpL3_{1\text{-}753}$ model, are indicated — grey and blue rectangles denote non-transmembrane and transmembrane helices (TMs) respectively, and yellow arrows β-strands. Unresolved regions are shown by a dashed grey line and the C-terminal truncation at D753 is annotated. Residues conserved in all aligned orthologs are enclosed in red boxes. **(B)** Cartoon representation of *Mtb* $MmpL3_{1\text{-}753}$ colored by conservation, performed on the ConSurf server (Ashkenazy et al., 2016). Locations of the removed C-terminal domain (CTD) and unresolved TM6-7 loop are labelled. **(C)** & **(D)** ConSurf conservation of residues in the *Mtb* MmpL3 (C) TM6-7 loop (residues 343-377), and (D) CTD (residues 753-944); color-coded as in (B).

	Mtb MmpL3 (EMD-12604) (PDB: 7NVH)
**Data Collection & Processing**	
Magnification	105,000
Voltage (kV)	300
Electron Exposure (e-/Å^2)	58.2
Defocus Range (µm)	-3 to -0.5
Pixel Size (Å)	0.832
Symmetry Imposed	C1
Initial Particle Images (no.)	7,764,838
Final Particle Images (no.)	414,082
Map Resolution (Å)	3.0
FSC Threshold	0.143
Map Resolution Range (Å)	3.0 – 4.0
**Refinement**	
Initial Model Used (PDB Code)	6OR2
Model Resolution (Å)	3.0
FSC Threshold	0.143
Model Resolution Range (Å)	3.0 – 4.0
Map Sharpening *B* factor (Å^2)	-86.7
Model Composition	
Non-hydrogen Atoms	5529
Protein Residues	717
Ligands	1
B Factors (Å^2)	
Protein	53.19
Ligand	63.35
Root-mean-square Deviations	
Bond Lengths (Å)	0.004
Bond Angles (°)	0.641
Validation	
MolProbity Score	1.78
Clashscore	7.31
Poor Rotamers (%)	0.69
Ramachandran Plot	
Favoured (%)	94.39
Allowed (%)	5.61
Disallowed (%)	0

Table S1. Cryo-EM Data Collection, Refinement and Model Validation Statistics, Related to Figure 1
